# Synthesis of New 2-Halo-2-(1*H-*tetrazol-5-yl)-2*H*-azirines via a Non-Classical Wittig Reaction

**DOI:** 10.3390/molecules201219848

**Published:** 2015-12-14

**Authors:** Ana L. Cardoso, Carmo Sousa, Marta S. C. Henriques, José A. Paixão, Teresa M. V. D. Pinho e Melo

**Affiliations:** 1 Centro de Química de Coimbra and Department of Chemistry, University of Coimbra, Coimbra 3004-535, Portugal; analcclopes@gmail.com (A.L.C.); carmo.b.sousa@gmail.com (C.S.); 2 CFisUC and Department of Physics, University of Coimbra, Coimbra P-3004-516, Portugal; marta.henriques@gmail.com (M.S.C.H.); jap@fis.uc.pt (J.A.P.)

**Keywords:** 2-halo-2*H*-azirines, vinyl tetrazoles, tetrasubstituted alkenes, phosphorus ylides

## Abstract

The synthesis and reactivity of tetrazol-5-yl-phosphorus ylides towards *N*-halosuccinimide/TMSN_3_ reagent systems was explored, opening the way to new haloazidoalkenes bearing a tetrazol-5-yl substituent. These compounds were obtained as single isomers, except in one case. X-ray crystal structures were determined for three derivatives, establishing that the non-classical Wittig reaction leads to the selective synthesis of haloazidoalkenes with (*Z*)-configuration. The thermolysis of the haloazidoalkenes afforded new 2-halo-2-(tetrazol-5-yl)-2*H*-azirines in high yields. Thus, the reported synthetic methodologies gave access to important building blocks in organic synthesis, vinyl tetrazoles and 2-halo-2-(tetrazol-5-yl)-2*H*-azirine derivatives.

## 1. Introduction

2*H*-azirines are highly reactive and easily available compounds. Thus, they have been widely used as versatile building blocks for the synthesis of various nitrogen-containing compounds. They can act as nucleophiles, electrophiles, dienophiles, and dipolarophiles in a variety of organic reactions. Furthermore, selective cleavage of each of the three bonds can be achieved, and this leads to highly reactive intermediates such as vinylnitrenes, nitrile ylides, and iminocarbenes [1,2,3,4,5,6].

We have previously described a general route to tetrasubstituted alkenes via a non-classical Wittig reaction [7]. Particularly interesting was the possibility of preparing haloazidoalkenes since the study of their thermolysis led to the development of a new route to 2-halo-2*H*-azirines starting from α-oxophosphorus ylides [8,9,10]. This study allowed the synthesis of a range of 2-halo-2*H*-azirines with several substituents, including the first examples of 2-bromo and 2-iodo-2*H*-azirine derivatives. Since then, a few examples of halo substituted azirines prepared from haloazidoalkenes by thermal or photochemical decomposition have been reported [11,12,13,14].

The study of the reactivity of these compounds showed that they can be used to prepare other functionalized 2*H*-azirines [15,16], but they can also lead to the synthesis of other interesting structures such as quinoxalines [15], functionalized 1,3-oxazoles [17,18], 1-aminovinyl derivatives [16], 4-halo-2-azabuta-1,3-dienes, and 2,3-dihydroazetes [19,20].

Recently, we became interested in the development of synthetic routes to functionalized 5-(substituted)-1*H*-tetrazoles. In this context, the synthesis of novel 2-(tetrazol-5-yl)-2*H*-azirines using the Neber approach, has been reported [21]. We envisaged that these three-membered heterocyclic compounds could be particularly interesting as building blocks for the synthesis of new 5-substituted tetrazoles. In fact, their reactivity towards imines was studied resulting in a novel and efficient route to 4-(tetrazol-5-yl)-1*H*-imidazoles, a class of compounds with potential biological activity [22]. Aiming to extend this approach to 5-substituted tetrazoles, we decided to prepare 2*H*-azirines combining halogen and tetrazole functionalities, since the presence of the extra functional group could be particularly interesting.

**Scheme 1:**
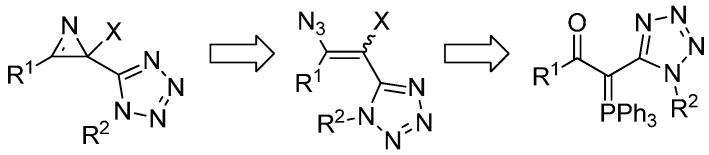
Synthetic strategy for the synthesis 2-halo-2-(1*H*-tetrazol-5-yl)-2*H*-azirines.

Using the synthetic methodology previously developed in our group for the preparation of 2-halo-2*H*-azirines from phosphorus ylides, we carried out reactivity studies of α-oxophosphorus ylides bearing a tetrazole substituent towards *N*-halossucinimides/TMSN_3_ reagent systems followed by thermolysis of the corresponding haloazidoalkenes (Scheme 1).

## 2. Results and Discussion

**Scheme 2:**
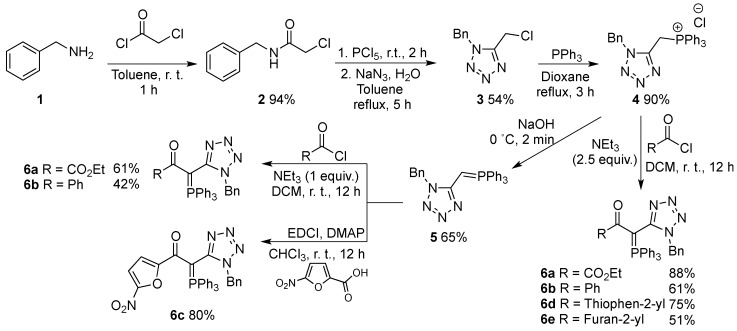
Synthesis of tetrazol-5-yl phosphorus ylides **6**.

The synthesis of the target tetrazol-5-yl phosphorus ylides **6** is outlined in Scheme 2. *N*-Benzylchloroacetamide (**2**) was prepared in good yield from the reaction of benzylamine and chloroacetyl chloride by an analogous method to that described in the literature [23]. Chloroacetamide **2** was treated with phosphorus pentachloride, followed by addition of sodium azide and water to give 1-benzyl-5-chloromethyltetrazole (**3**) in 54% yield [24]. Reaction of chloromethyltetrazole **3** with triphenylphosphine afforded the corresponding phosphonium salt **4** in very high yield (90%), which was subsequently neutralized with aqueous sodium hydroxide solution over a short period of time with ice-cooling to give phosphorus ylide **5** bearing a tetrazolyl substituent in moderate yield (65%). As previously observed with other tetrazolic phosphorus ylides, phosphorane **5** was hydrolyzed in water to give triphenylphosphine oxide and 5-methyl-1*H*-tetrazole [25,26]. For this reason, in order to prevent this hydrolysis the base treatment of **4** was carried out in water for only 2 min with vigorous stirring and the resulting precipitate was filtered and immediately dried under reduced pressure. However, even with these controlled conditions mixtures of ylide and hydrolysis products were obtained making the purification procedure difficult.

Reaction of phosphorus ylide **5** with ethyl oxalyl chloride and benzoyl chloride in the presence of triethylamine gave ylides **6a** and **6b**, respectively, in moderate yields (Scheme 2). Aiming to improve ylides of **6a** and **6b** yield and to overcome the difficulties observed in the synthesis of ylide **5**, we tried to carry out the synthesis of ylides **6** starting directly from the phosphonium salt **4** in the presence of triethylamine. To our delight, carrying out the reaction of the phosphonium salt **4** with ethyl oxalyl chloride and benzoyl chloride in the presence of excess of triethylamine led to the formation of ylides **6a** and **6b** in 88% and 61% yield, respectively. The same methodology was applied to the synthesis of ylides **6d** and **6e** bearing a thiophenyl and a furanyl substituent, respectively, which were isolated in good yields (Scheme 2). On the other hand, reaction of ylide **5** with 5-nitro-furan-2-carboxylic acid in the presence of 1-ethyl-3-(3-dimethylaminopropyl)carbodiimide (EDCI) and 4-dimethylaminopyridine (DMAP) afforded ylide **6c** in high yield (80%).

**Scheme 3:**
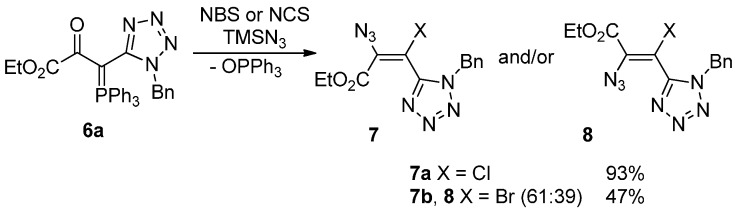
Reactivity of phosphorus ylide **6a** towards *N*-halosuccinimides in the presence of TMSN_3_.

**Scheme 4:**
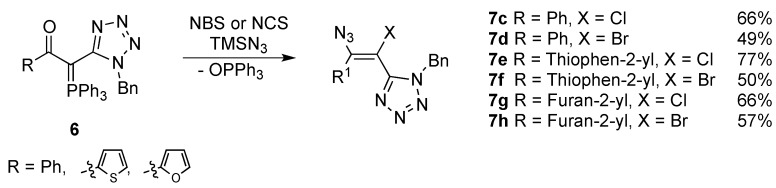
Reactivity of phosphorus ylides **6b**, **6d** and **6e** towards NXS/TMSN_3_ reagent system.

These ylides reacted with *N*-halosuccinimides in the presence of azidotrimethylsilane giving the corresponding haloazidoalkenes **7a**–**h** and **8** in yields ranging from 47% to 93% (Scheme 3 and Scheme 4). Higher yields were obtained when NCS/TMSN_3_ were used as reagents in the reactions with all ylides **6**. The reaction of NCS with ylide **6a** in the presence of TMSN_3_ led to the formation of the desired chloroazidoalkene **7a** with the highest yield (93%). As for bromoazidoalkenes, the best result was obtained from the reaction of ylide **6e** bearing a furanyl substituent with NBS/TMSN_3_ reagent system which led to the formation of the corresponding bromoazidoalkene **7h** in 57% yield. The reactions with *N*-chlorosuccinimide were completed after 1–1.5 h while the reactions with *N*-bromosuccinimide required longer periods of time (2–3 h). The azidoalkenes were obtained selectively as single isomers except in the case of **7b** and **8** which was obtained as a mixture of *E* and *Z* isomers (61:39).

**Figure 1:**
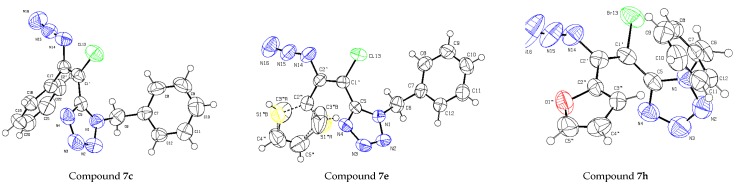
X-ray structures of compounds **7c**, **7e** and **7h**.

**Figure 2:**
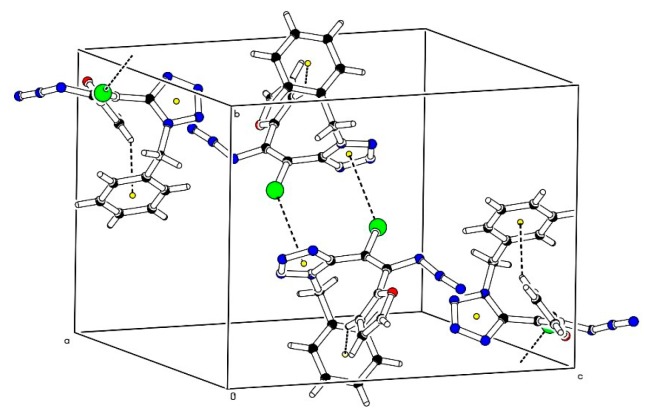
Crystal packing of **7h** showing the network of C–H···Cg and Br–Cg interactions.

In order to establish the stereochemistry of the synthetized alkenes compounds **7c**, **7e** and **7h**, bearing a phenyl group, a thiophenyl group and a furanyl group at C-2′, respectively, were selected for X-ray crystallography studies. The three compounds crystallize in the same, monoclinic, space group (*P*2_1_/*c*). The X-ray data unambiguously shows that the molecules adopt in the crystal the (*Z*)-configuration (Figure 1). Although there is a significant freedom for rotation of substituents around single C–C bonds, no sign for disorder was found except for the thiophene ring in compound **7e**, which features a minor disorder between two alternating positions related by a 180° rotation around the C2′′–C2′ bond with occupancies 67:33%. A selection of bond distance, bond angles and torsion angles is provided in Table 1. They are in agreement with typical average values and also to those of the XRD study of a bromo-azidoalkene reported in [17]. Cohesion of the crystal structures is provided by weak C–H···N hydrogen bonds and also C–H···Cg, Cg···Cg and Br···Cg interactions involving the aromatic rings (Figure 2).

**Table 1:** Selected bond distances (Å), bond angles (°) and torsion angles (°) for compounds **7c**, **7h** and **7e**. Atom X is either Cl (**7c** and **7e**) or Br (**7h**).

Bond Distances or Angles	7c	7h	7e
C1′–C2′	1.331(3)	1.333(5)	1.334(4)
C2′–N14	1.410(3)	1.409(4)	1.407(4)
C1′–X13	1.727(2)	1.889(3)	1.718(3)
C1′–C5	1.460(4)	1.448(5)	1.458(2)
N14–C2′–C1′	116.9(2)	118.4(3)	116.9(3)
C2′–C1′–X	122.5(2)	120.6(3)	122.0(2)
C2′–C1′–C5	122.7(2)	124.8(3)	122.9(2)
C5–C1′–X	114.9(2)	114.5(2)	114.9(2)
N14–C2′–C1′–C5	−179.0(2)	−170.0(3)	171.1(3)
N14–C2′–C1′–X	0.2(3)	5.9(4)	−3.4(4)
C5–N1–C6–C7	94.2(3)	74.4(5)	100.7(3)
N1–C6–C7–C8	−105.3(3)	−104.6(4)	−117.7(3)
X–C1′–C5–N1	77.5(3)	66.5(4)	−71.6(3)
C2′–C1′–C5–N1	76.1(3)	−117.4(4)	113.4(3)

Since **7d**, **7f** and **7g** differ from **7c**, **7e** and **7h** only in the nature of the halogen, the (*Z*)-configuration is therefore proposed for all of these compounds. In previous studies, we could confirm that our synthetic methodology allowed the synthesis of a bromoazidoalkene bearing a carboxylate group at C-1′ and a phenyl group at C-2′ with the same selectivity [17]. Thus, the stereochemistry outcome is retained when the carboxylate group is replaced by a tetrazolyl group (**7c**).

**Scheme 5:**
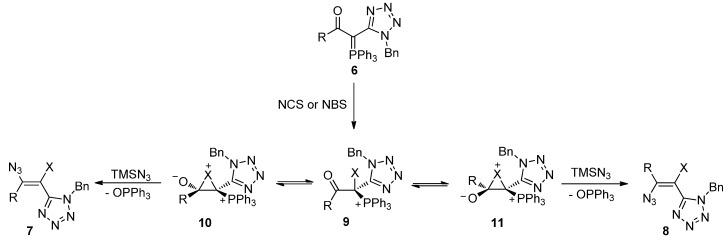
Formation of isomeric halonium ions as intermediates of the reaction.

The synthesis of the haloazidoalkenes can be rationalized as outlined in Scheme 5. The formation of the observed products can be explained by considering isomeric halonium ions **10** and **11** as intermediates. These halonium ions can interconvert by way of acyclic cation **9**. The opening of these intermediates by the TMSN_3_ leads to the isomeric alkenes after the elimination of triphenylphosphine oxide (Scheme 5). The observed selected formation of alkenes with (*Z*) configuration may result from the higher stability of halonium ion **10** in comparison with the isomeric intermediate **11**.

**Scheme 6:**
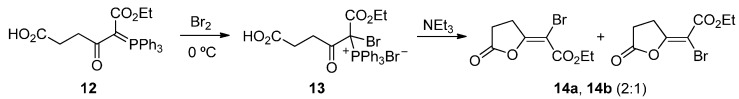
Synthesis of halogenated enol lactones from keto acid phosphoranes.

The formation of halophosphonium salt **9** is the expected intermediate of the halogenation of α-oxophosphorus ylides, which affords the corresponding halophosphonium salts [27]. Moreover, the synthesis of halogenated enol lactones from keto acid phosphoranes via an intramolecular non-classical Wittig reaction has also been described [28,29,30,31]. In fact, α-oxophosphorus ylides bearing a terminal carboxylic acid group react with halogenating agents leading to *E*- and *Z*-halo enol lactones. This cyclization was rationalized via a halophosphonium salt followed by loss of triphenylphosphine oxide. Indeed, bromophosphonium salt **13** could be isolated from the reaction of ylide **12** with bromine at 0 °C in the absence of NEt_3_. Treatment of **13** with triethylamine leads to the corresponding bromo enol lactones **14** (Scheme 6) [28].

The ^13^C-NMR spectra of the haloazidoalkenes **7a**–**h** show the C–X carbon between 85.8 and 109.3 ppm and the C-N_3_ between 134.8 and 147.6 ppm (Table 2). As expected, the chemical shift of C–X carbon of all bromoazidoalkenes is lower than the ones of the corresponding chloroazidoalkenes (e.g., **7b** *vs.* **7a**).

**Table 2:** ^13^C NMR in CDCl_3_ of the haloazidoalkenes **7** and 2*H*-Azirines **15** (δ in ppm).

Alkene	C–X	C–N_3_	2*H*-Azirine	C-2	C-3
**7a**	109.3	135.3	**15a**	51.9	156.8
**7b**	97.8	137.3	**15b**	40.7	157.4
**7c**	98.5	145.4	**15c**	46.4	168.6
**7d**	85.8	147.6	**15d**	33.8	169.6
**7e**	100.4	138.6	**15e**	47.0	161.7
**7f**	87.9	140.7	**15f**	34.5	162.7
**7g**	99.8	134.8	**15g**	46.1	157.7
**7h**	87.2	136.8	**15h**	33.3	158.5

**Scheme 7:**
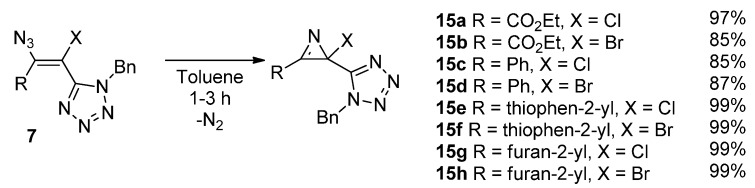
Synthesis of 2-halo-2-(tetrazol-5-yl)-2*H*-azirines **15**.

The thermolysis of the haloazidoalkene derivatives **7** was then investigated (Scheme 7). Initially, attempts were made to promote these reactions in *n*-heptane. However, due to the low solubility of the haloazidoalkenes in this solvent, the thermolysis in *n*-heptane often led to complex mixtures of the desired 2*H*-azirines and degradation products. Nonetheless, carrying out the reaction of these haloazidoalkenes in toluene at 90 °C for 2–3 h led efficiently to the formation of new 2-halo-2-tetrazol-5-yl-2*H*-azirines **15**. The reaction can be followed by TLC and by IR by monitoring the disappearance of the band corresponding to the azido group of the starting azidoalkenes (ν ~2105–2130 cm^−1^). Regardless of C-3 substituents, 2-bromo- and 2-chloro-2*H*-azirines **15** were obtained in high yield (85%–99%).

The ^13^C-NMR spectra of the 2-chloro- and 2-bromo-2-(tetrazol-5-yl)-2*H*-azirines **15** show the sp^2^ carbon between 156.8 and 169.6 ppm and the sp^3^ carbon between 33.3 and 51.9 ppm, depending on the substitution pattern (Table 2).

**Scheme 8:**
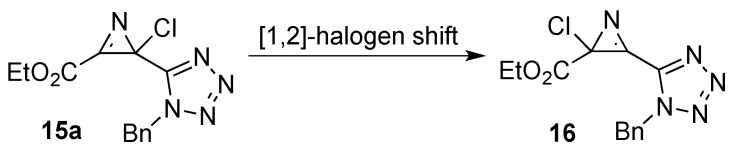
Isomerization of 2-halo-2-(tetrazol-5-yl)-2*H*-azirine **15a**.

It is well established that some 2-halo-2*H*-azirines undergo thermal rearrangement to their azirine isomers through a [1,2]-halogen shift [32,33]. Recently, Banert *et al*. reported optimized reaction conditions to favor the complete and irreversible isomerization of 2-halo-2*H*-azirines [11]. In our case, it was possible to isolate 2*H*-azirines **15** as pure isomers by thermolysis of the haloazidoalkenes **7**. However, after being stored at −30 °C for 3 months 2-chloro-2*H*-azirine **15a**, bearing a carboxylate group at C-3, underwent rearrangement to a mixture of 2*H*-azirines **15a** and **16** (Scheme 8). Carrying out NMR measurements at different temperatures (25–95 °C), the variation of the isomer ratio with increasing temperature was observed, until complete rearrangement of 2*H*-azirine **15a** into the isomer **16a** (Supplementary Materials). Similar NMR experiments with **15c** and **15e** did not indicate the same behavior.

## 3. Experimental Section

### 3.1. General Information

NMR spectra were run in CDCl_3_ or DMSO-*d*_6_ on a 400 MHz Bruker Avance III spectrometer (Bruker Biospin SA, Wissembourg, France) and recorded at the following frequencies: proton (^1^H, 400 MHz), carbon (^13^C, 100 MHz). Chemical shifts are expressed in parts per million related to internal TMS and coupling constants (*J*) are in hertz. Infrared spectra (IR) were recorded on a Nicolet 6700 FTIR spectrometer (Thermo Scientific, Waltham, MA, USA). Mass spectra were recorded in electrospray ionization (ESI) mode on a Bruker FTMS APEX III spectrometer (Bruker Corporation, Bremen, Germany). Melting points were determined in open glass capillaries and are uncorrected. Thin-layer chromatography (TLC) analyses were performed using precoated silica gel plates (Merck KGaA, Darmstadt, Germany). Flash column chromatography was performed with silica gel 60 as the stationary phase.

### 3.2. Experimental Details

#### 3.2.1. Synthesis of Phosphorus Ylide **5**

*N-Benzyl-2-chloroacetamide* (**2**): Chloroacetamide **2** was prepared following the procedure described in the literature [23]. To a stirred solution of benzylamine (7.8 mL, 70.8 mmol) in toluene (60 mL) under cooling with ice bath, chloroacetyl chloride (4 g, 35.4 mmol) was slowly added. The reaction mixture was stirred vigorously for 1h at room temperature. The solvent was evaporated under vacuum, the crude reaction was dissolved in dichloromethane (100 mL) and washed with water (3 × 50 mL). The organic layer was dried over anhydrous MgSO_4_, filtered and the solvent evaporated under vacuum. The product was obtained as a white solid (6.30 g, 97%). m.p. 91–92 °C (93–96 °C from literature) [23]; ^1^H-NMR (CDCl_3_) *δ* 4.11 (s, 2H), 4.50 (d, 2H, *J* = 6.0 Hz), 6.89 (br s, 1H), 7.26–7.36 (m, 5H, Ar-H).

*1-Benzyl-5-(chloromethyl)-1H-tetrazole* (**3**): Compound **3** was prepared by an analogous method to that described in the literature [24]. PCl_5_ (7.06 g, 33.9 mmol) was added slowly to a solution of *N*-benzyl-2-chloroacetamide (5.66 g, 30.8 mmol) in toluene (50 mL) under cooling with ice-water bath. The mixture was stirred at room temperature for 2 h, then NaN_3_ (3.01 g, 46.3 mmol) was added. The reaction mixture was stirred at room temperature for 30 min, water (0.8 mL) was added dropwise and the whole was refluxed for 5 h. After cooling, the reaction mixture was poured into water and extracted with chloroform. The combined organic layers were washed successively with water, NaOH solution 1M and saturated NaCl solution and dried over anhydrous MgSO_4_. After removal of the solvent, the crude product was purified by flash chromatography (ethyl acetate/hexane (1:2)) affording the tetrazole **3** as light yellow solid (3.47 g, 54%). m.p. 57–59 °C (from diethyl ether) (62–63 °C from literature) [24]; ^1^H-NMR (CDCl_3_) δ (ppm) 4.62 (s, 2H), 5.68 (s, 2H), 7.28–7.30 (m, 2H, Ar-H), 7.39–7.40 (m, 3H, Ar-H).

*((1-Benzyl-1H-tetrazol-5-yl)methyl)triphenylphosphonium chloride* (**4**): Compound **4** was prepared by an analogous method to that described in the literature [26]. A solution of PPh_3_ (1.43 g, 5.47 mmol) and 1-benzyl-5-(chloromethyl)-1*H*-tetrazole (1.14 g, 5.47 mmol) in dioxane (10 mL) was refluxed for 3 h. Phosphonium salt **4** precipitates and is filtered and washed with Et_2_O. White solid (2.32 g, 90%). m.p. > 257 °C (decomp., from diethyl ether); IR (KBr) 490, 523, 688, 722, 1113, 1170, 1434, 1494 cm^−1^; ^1^H-NMR (CDCl_3_) δ (ppm) 5.92 (d, *J* = 14. 4 Hz, 2H), 6.33 (s, 2H), 7.26–7.29 (m, 3H, Ar-H), 7.53–7.63 (m, 8H, Ar-H), 7.74–7.85 (9H, m, Ar-H); ^13^C-NMR (CDCl_3_) δ (ppm) 21.7 (d, ^1^*J*_CP_ = 56.9 Hz), 51.5, 117.5 (d, ^1^*J*_CP_ = 88.6 Hz), 128.7, 128.8, 129.1, 130.2, 130.3, 133.6, 134.1, 134.2, 135.3, 146.4 (d, ^2^*J*_CP_ = 3.9 Hz).

*1-Benzyl-5-((triphenylphosphoranylidene)methyl)-1H-tetrazole* (**5**): The phosphonium salt **4** (1.0 g, 2.13 mmol) was dissolved in a mixture of H_2_O (22 mL) and MeOH (6 mL) and cooled on ice bath. A solution of NaOH (85 mg, 2.13 mmol) in H_2_O (2 mL) was added over 1 min with vigorous stirring. The mixture was stirred for 1 min and filtered. The precipitate was washed with cold H_2_O and immediately dried under reduced pressure affording the ylide **5** as a light yellow solid (0.83 g, 90%). m.p. 243–245 °C (from diethyl ether); IR (KBr) 489, 506, 522, 686, 719, 1112, 1434 cm^−1^; ^1^H-NMR (CDCl_3_) δ (ppm) 4.29 (d, *J* = 6.4 Hz, 1H), 5.12 (d, *J* = 14.4 Hz, 2H), 7.17–7.19 (m, 3H, Ar-H), 7.28–7.29 (m, 3H, Ar-H), 7.57–7.60 (m, 7H, Ar-H), 7.73–7.80 (7H, m, Ar-H); ^13^C-NMR (CDCl_3_) δ (ppm) 32.1 (d, ^1^*J*_CP_ = 54.9 Hz), 43.6, 118.4 (d, ^1^*J*_CP_ = 88.0 Hz), 126.8, 128.0, 128.3, 128.4, 128.6, 128.9, 130.0, 130.1, 132.0, 132.1, 134.0, 134.1, 134.9, 138.0, 162.5 (d, ^2^*J*_CP_ = 4.9 Hz); HRMS (ESI) calcd for C_27_H_24_N_4_P 435.1733 [M + H]^+^, found 435.1738.

#### 3.2.2. General Procedure for the Synthesis of Ylides **6**

A solution of phosphonium salt **4** (10 mmol) and triethylamine (2.53 g, 25 mmol) in dry CHCl_3_ (50 mL) was stirred at room temperature while a solution of the appropriate acid chloride (12 mmol) in dry CHCl_3_ (10 mL) was added dropwise to it. After the addition, the mixture was stirred at room temperature for 12 h. The reaction mixture was washed with H_2_O (3 × 50 mL), dried and evaporated to give the desired ylides **6** which were recrystallized from ethyl acetate.

*Ethyl 3-(1-benzyl-1H-tetrazol-5-yl)-2-oxo-3-(triphenylphosphoranylidene)propanoate* (**6a**): Ylide **6a** was obtained as a light yellow solid (4.70 g, 88%). m.p. 174-176 °C (from ethyl acetate/ hexane); IR (KBr) 524, 557, 696, 1103, 1193, 1436, 1540, 1735 cm^−1^; ^1^H-NMR (CDCl_3_) δ (ppm) 1.10 (t, *J* = 7.2 Hz, 3H), 4.02 (q, *J* = 7.2 Hz, 2H), 5.43 (br s, 1H), 5.51 (br s, 1H), 7.11–7.13 (m, 2H, Ar-H), 7.21–7.23 (m, 3H, Ar-H), 7.36–7.41 (m, 11H, Ar-H), 7.53–7.56 (4H, m, Ar-H); ^13^C-NMR (CDCl_3_) δ (ppm) 13.9, 51.0, 57.6 (d, ^1^*J*_CP_ = 114 Hz), 61.5, 123.0 (d, ^1^*J*_CP_ = 92.0 Hz), 128.4, 128.5, 128.6, 128.7, 128.9, 129.1, 132.9, 133.6, 133.7, 133.8, 134.1, 134.2, 151.9 (d, ^2^*J*_CP_ = 12.6 Hz), 164.4 (d, ^3^*J*_CP_ = 15.2 Hz), 175.0 (d, ^2^*J*_CP_ = 6.2 Hz); HRMS (ESI) calcd for C_31_H_28_N_4_O_3_P 535.18935 [M + H]^+^, found 535.18932.

*2-(1-Benzyl-1H-tetrazol-5-yl)-1-phenyl-2-(triphenylphosphoranylidene)ethanone* (**6b**): Ylide **6b** was obtained as a white solid (2.75 g, 61%). m.p. 213-214 °C (from ethyl acetate/ hexane); IR (KBr) 507, 693, 723, 1095, 1102, 1337, 1434, 1529 cm^−1^; ^1^H-NMR (CDCl_3_) δ (ppm) 4.70 (br s, 1H), 4.94 (br s, 1H), 6.95–6.96 (m, 2H, Ar-H), 7.01–7.04 (m, 2H, Ar-H), 7.08–7.14 (m, 3H, Ar-H), 7.19–7.32 (m, 9H, Ar-H), 7.37–7.46 (9H, m, Ar-H); ^13^C-NMR (CDCl_3_) δ (ppm) 50.3, 55.7 (d, ^1^*J*_CP_ = 120.4 Hz), 124.9 (d, ^1^*J*_CP_ = 92.5 Hz), 127.2, 128.3, 128.4, 128.6, 128.7, 128.8, 129.7, 132.4, 133.6, 133.7, 133.8, 141.0 (d, ^3^*J*_CP_ = 9.9 Hz), 153.3 (d, ^2^*J*_CP_ = 15.9 Hz), 186.0 (d, ^2^*J*_CP_ = 4.6 Hz); HRMS (ESI) calcd for C_34_H_28_N_4_OP 539.1995 [M + H]^+^, found 539.1997.

*2-(1-Benzyl-1H-tetrazol-5-yl)-1-(thiophen-2-yl)-2-(triphenylphosphoranylidene)ethanone* (**6d**): Ylide **6d** was obtained as a white solid (4.08 g, 75%). m.p. > 210 °C (decomp., from ethyl acetate/ hexane); IR (KBr) 531, 691, 715, 1106, 1352, 1503 cm^−1^; ^1^H-NMR (CDCl_3_) δ (ppm) 5.09 ( br d, *J* = 12.4 Hz, 1H), 5.33 ( br d, *J* = 12.4 Hz, 1H), 6.37 (dd, *J* = 1.0 Hz and *J* = 3.6 Hz, 1H), 6.80 (dd, *J* = 4.0 Hz and *J* = 4.8 Hz, 1H), 7.06–7.10 (m, 2H, Ar-H), 7.14–7.19 (m, 3H, Ar-H), 7.29 (d, *J* = 4.4 Hz, 1H), 7.34–7.39 (m, 6H, Ar-H), 7.42–7.47 (m, 6H, Ar-H), 7.50–7.54 (m, 3H, Ar-H); ^13^C-NMR (CDCl_3_) δ (ppm) 50.7, 53.7 (d, ^1^*J*_CP_ = 122.7 Hz), 124.7 (d, ^1^*J*_CP_ = 92.6 Hz), 127.4, 127.7, 128.5, 128.6, 128.8, 128.9, 132.4, 133.5, 133.7, 133.8, 145.4 (d, ^3^*J*_CP_ = 12.0 Hz), 152.8 (d, ^2^*J*_CP_ = 15.0 Hz), 177.4 (d, ^2^*J*_CP_ = 5.9 Hz); HRMS (ESI) calcd for C_32_H_26_N_4_OPS 545.1559 [M + H]^+^, found 545.1548.

*2-(1-Benzyl-1H-tetrazol-5-yl)-1-(furan-2-yl)-2-(triphenylphosphoranylidene)ethanone* (**6e**): Ylide **6e** was obtained as a white solid (2.70 g, 51%). m.p. > 220 °C (decomp., from ethyl acetate/ hexane); IR (KBr) 522, 689, 721, 1106, 1456, 1512 cm^−1^; ^1^H-NMR (CDCl_3_) δ (ppm) 5.20 ( br d, *J* = 14.4 Hz, 1H), 5.43 ( br d, *J* = 14.4 Hz, 1H), 6.26 (dd, *J* = 1.6 Hz and *J* = 3.6 Hz, 1H), 6.40 (d, *J* = 2.8 Hz, 1H), 7.08–7.12 (m, 3H, Ar-H), 7.17–7.21 (m, 3H, Ar-H), 7.34–7.39 (m, 6H, Ar-H), 7.43–7.48 (m, 6H, Ar-H), 7.52–7.54 (m, 3H, Ar-H); ^13^C-NMR (CDCl_3_) δ (ppm) 50.6, 53.4 (d, ^1^*J*_CP_ = 122.5 Hz), 111.2, 113.0, 124.6 (d, ^1^*J*_CP_ = 92.6 Hz), 128.4, 128.6, 128.7, 128.8, 128.9, 132.4, 132.5, 133.5, 133.6, 143.6, 152.6 (d, ^3^*J*_CP_ = 13.9 Hz), 153.2 (d, ^2^*J*_CP_ = 12.2 Hz), 173.8 (d, ^2^*J*_CP_ = 5.7 Hz). HRMS (ESI) calcd for C_32_H_26_N_4_O_2_P 529.1787 [M + H]^+^, found 529.1784.

*2-(1-Benzyl-1H-tetrazol-5-yl)-1-(5-nitrofuran-2-yl)-2-(triphenylphosphoranylidene)ethanone* (**6c**): Compound **6c** was prepared by an analogous method to that described in the literature [34]. A solution of phosphorus ylide **5** (2.1 mmol) and 5-nitrofuran-2-carboxylic acid (2.5 mmol) in dry CHCl_3_ (40 mL) was cooled in an ice bath. Then EDCI (3.2 mmol) and DMAP (catalytic) was added to it. After the addition, the mixture was stirred at room temperature for 12 h. The reaction mixture was washed with H_2_O (3 × 50 mL), dried and evaporated. The crude product was purified by flash chromatography (ethyl acetate). Ylide **6c** was obtained as a yellow solid (4.59 g, 80%). m.p. > 210 °C (decomp., from ethyl acetate/ hexane); IR (KBr) 515, 525, 688, 1099, 1300, 1541 cm^−1^; ^1^H-NMR (CDCl_3_) δ (ppm) 5.38 (d, *J* = 14.8 Hz, 1H), 5.49 (d, *J* = 14.8 Hz, 1H), 6.60 (d, *J* = 4.0 Hz, 1H), 6.80 (d, *J* = 4.0 Hz, 1H), 7.09–7.12 (m, 2H, Ar-H), 7.17–7.24 (m, 3H, Ar-H), 7.42–7.43 (m, 6H, Ar-H), 7.48–7.56 (m, 6H, Ar-H), 7.58–7.59 (m, 3H, Ar-H); ^13^C-NMR (CDCl_3_) δ (ppm) 50.9, 56.3 (d, ^1^*J*_CP_ = 118.2 Hz), 111.6, 114.4, 123.4 (d, ^1^*J*_CP_ = 92.4 Hz), 128.5, 128.6, 129.1, 129.2, 133.0, 133.4, 133.5, 133.6, 150.9, 151.6, 153.4 (d, ^2^*J*_CP_ = 12.3 Hz), 171.2 (d, ^2^*J*_CP_ = 6.6 Hz); HRMS (ESI) calcd for C_32_H_25_N_5_O_4_P 574.1638 [M + H]^+^, found 574.1631.

#### 3.2.3. General Procedure for the Synthesis of Haloazidoalkenes **7** and **8**

Ylide **6** (4.5 mmol) was dissolved in dichloromethane (50 mL) and a solution of azidotrimethylsilane (0.71 g, 6.5 mmol) and *N*-chloro- or *N*-bromosuccinimide (6.5 mmol) in dichloromethane (10 mL) was added. The reaction mixture was stirred at room temperature for the appropriate time (1–3 h). After removal of the solvent, the crude product was purified by flash chromatography (ethyl acetate/hexane).

*Ethyl 2-azido-3-(1-benzyl-1H-tetrazol-5-yl)-3-chloropropenoate* (**7a**): Haloazidoalkene was obtained as a white solid (1.41 g, 93%). m.p. 87.4–88.1 °C (from ethyl acetate/ hexane); IR (KBr) 699, 722, 1232, 1258, 1723, 2119 cm^−1^; ^1^H-NMR (CDCl_3_) δ (ppm) 0.80 (t, *J* = 7.2 Hz, 3H), 3.84 (q, *J* = 7.2 Hz, 2H), 5.50 (s, 2H), 7.34–7.37 (m, 5H, Ar-H); ^13^C-NMR (CDCl_3_) δ (ppm) 13.1, 52.0, 63.4, 109.3, 128.7, 129.0, 129.2, 129.3, 132.3, 135.3, 150.5, 158.8; HRMS (ESI) calcd for C_13_H_13_ClN_7_O_2_ 334.0813 [M + H]^+^, found 334.0813.

*Ethyl 2-azido-3-(1-benzyl-1H-tetrazol-5-yl)-3-bromopropenoate (***7b** *and* **8b***)*: Haloazidoalkenes were obtained as a mixture of isomers (61:39), yellow solid (0.71 g, 47%). IR (KBr) 696, 721, 1232, 1255, 1719, 2117 cm^−1^. ^1^H-NMR (CDCl_3_) δ (ppm) *Major isomer* 0.78 (t, *J* = 7.2 Hz, 3H), 3.80 (q, *J* = 7.2 Hz, 2H), 5.48 (s, 2H), 7.33–7.38 (m, 5H, Ar-H); *Minor isomer* 1.43 (t, *J* = 7.2 Hz, 3H), 4.46 (q, *J* = 7.2 Hz, 2H), 5.53 (s, 2H), 7.26–7.36 (m, 5H, Ar-H); ^13^C-NMR (CDCl_3_) δ (ppm) *Major isomer* 13.1, 52.0, 63.4, 97.8, 128.8, 129.0, 129.3, 132.3, 137.3, 151.6, 158.4; *Minor isomer* 14.0, 53.2, 64.5, 94.1, 128.3, 128.4, 128.7, 129.2, 129.4, 129.8, 136.7, 150.6, 159.7; HRMS (ESI) calcd for C_13_H_13_BrN_7_O_2_ 378.0308 [M + H]^+^, found 378.0307.

*(Z)-5-(2-Azido-1-chloro-2-phenylvinyl)-1-benzyl-1H-tetrazole* (**7c**): Haloazidoalkene was obtained as a white solid (1.00 g, 66%). m.p. 90.7-92.1 °C (from ethyl acetate/ hexane); IR (KBr) 699, 715, 1203, 1317, 1628, 2105, 2113 cm^−1^; ^1^H-NMR (CDCl_3_) δ (ppm) 5.41 (s, 2H), 6.81 (d, *J* = 7.2 Hz, 2H), 7.18–7.23 (m, 4H, Ar-H), 7.30–7.32 (m, 1H, Ar-H), 7.36–7.39 (m, 3H, Ar-H); ^13^C-NMR (CDCl_3_) δ (ppm) 51.6, 98.5, 128.2, 128.3, 129.0, 129.2, 129.7, 130.7, 132.8, 145.4, 150.6; HRMS (ESI) calcd for C_16_H_13_ClN_7_ 338.0915 [M + H]^+^, found 338.0922.

*5-(2-Azido-1-bromo-2-phenylvinyl)-1-benzyl-1H-tetrazole* (**7d**): Haloazidoalkene was obtained as a white solid (0.84 g, 49%). m.p. 88.8–90.0 °C (from ethyl acetate/hexane); IR (KBr) 698, 715, 1204, 1316, 1629, 2106, 2122 cm^−1^; ^1^H-NMR (CDCl_3_) δ (ppm) 5.38 (s, 2H), 6.82 (d, *J* = 7.2 Hz, 2H), 7.15–7.22 (m, 4H, Ar-H), 7.28–7.38 (m, 3H, Ar-H); ^13^C-NMR (CDCl_3_) δ (ppm) 51.5, 85.8, 128.1, 128.2, 129.0, 129.1, 129.2, 130.1, 130.7, 132.8, 147.6, 1515; HRMS (ESI) calcd for C_16_H_13_BrN_7_ 382.0410 [M + H]^+^, found 382.0418.

*(Z)-5-(2-Azido-1-chloro-2-(thiophen-2-yl)vinyl)-1-benzyl-1H-tetrazole* (**7e**): Haloazidoalkene was obtained as a light yellow solid (1.19 g, 77%). m.p. 83.4–84.2 °C (from ethyl acetate/hexane); IR (KBr) 526, 697, 712, 870, 1068, 1245, 1406, 1680, 2120 cm^−1^; ^1^H-NMR (CDCl_3_) δ (ppm) 5.39 (s, 2H), 6.76 (dd, *J* = 1.2 Hz and *J* = 3.6 Hz, 1H), 6.91 (dd, *J* = 3.6 Hz and *J* = 5.2 Hz, 1H), 7.20–7.22 (m, 2H, Ar-H), 7.33–7.39 (m, 4H, Ar-H); ^13^C-NMR (CDCl_3_) δ (ppm) 51.8, 1004, 127.7, 128.2, 129.1, 129.2, 129.9, 130.0, 130.4, 132.6, 138.6, 156.6; HRMS (ESI) calcd for C_14_H_11_ClN_7_S 344.0479 [M + H]^+^, found 344.0486.

*5-(2-Azido-1-bromo-2-(thiophen-2-yl)vinyl)-1-benzyl-1H-tetrazole* (**7f**): Haloazidoalkene was obtained as a white solid (0.77 g, 50%). m.p. 88.1–89.4 °C (from ethyl acetate/ hexane); IR (KBr) 694, 715, 798, 880, 1292, 1403, 1608, 2114 cm^−1^; ^1^H-NMR (CDCl_3_) δ (ppm) 5.36 (s, 2H), 6.77 (dd, *J* = 1.2 Hz and *J* = 3.6 Hz, 1H), 6.90 (dd, *J* = 3.6 Hz and *J* = 5.2 Hz, 1H), 7.18–7.21 (m, 2H, Ar-H), 7.33-7.36 (m, 4H, Ar-H); ^13^C-NMR (CDCl_3_) δ (ppm) 51.7, 87.9, 127.6, 128.3, 129.1, 129.8, 130.1, 130.3, 132.5, 140.7, 151.4; HRMS (ESI) calcd for C_14_H_11_BrN_7_S 387.9975 [M + H]^+^, found 387.9974.

*(Z)-5-(2-Azido-1-chloro-2-(furan-2-yl)vinyl)-1-benzyl-1H-tetrazole* (**7g**): Haloazidoalkene was obtained as a white solid (0.97 g, 66%). m.p. 72.3–72.7 °C (from ethyl acetate/ hexane); IR (KBr) 593, 687, 727, 804, 1018, 1321, 1624, 2132 cm^−1^; ^1^H-NMR (CDCl_3_) δ (ppm) 5.40 (s, 2H), 6.24 (d, *J* = 3.6 Hz, 1H), 6.29 (dd, *J* = 2.0 Hz and *J* = 3.6 Hz, 1H), 7.19–7.21 (m, 2H, Ar-H), 7.24 (d, *J* = 1.2 Hz, 1H), 7.31–7.32 (m, 3H, Ar-H); ^13^C-NMR (CDCl_3_) δ (ppm) 51.8, 99.8, 111.8, 114.4, 128.2, 129.0, 129.1, 132.5, 134.8, 145.0, 150.7; HRMS (ESI) calcd for C_14_H_11_ClN_7_O 328.0708 [M + H]^+^, found 328.0713.

*5-(2-Azido-1-bromo-2-(furan-2-yl)vinyl)-1-benzyl-1H-tetrazole* (**7h**): Haloazidoalkene was obtained as a light yellow solid (0.95 g, 57%). m.p. 84.0–85.3 °C (from ethyl acetate/hexane); IR (KBr) 697, 718, 1117, 1232, 1455, 1497, 2121 cm^−1^; ^1^H-NMR (CDCl_3_) δ (ppm) 5.36 (s, 2H), 6.21 (d, *J* = 3.6 Hz, 1H), 6.27 (dd, *J* = 2.0 Hz and *J* = 3.6 Hz, 1H), 7.16–7.19 (m, 2H, Ar-H), 7.24 (d, *J* = 1.2 Hz, 1H), 7.28–7.32 (m, 3H, Ar-H); ^13^C-NMR (CDCl_3_) δ (ppm) 51.7, 87.2, 111.8, 114.4, 128.2, 128.3, 128.9, 129.0, 129.2, 132.4, 136.7, 143.5, 144.8, 151.6; HRMS (ESI) calcd for C_14_H_11_BrN_7_O 372.0203 [M + H]^+^, found 372.0209.

#### 3.2.4. General Procedure for the Synthesis of 2-Chloro- and 2-Bromo-2*H*-azirines **15**

A solution of the vinyl azide **7** (2.0 mmol) in toluene (10 mL) was heated at 90 °C for 1–3 h (the reaction was monitored by TLC and reaction was complete when disappearance of the vinyl azide was observed).The reaction mixture was cooled and the solvent evaporated giving the 2*H*-azirine.

*Ethyl 2-(1-benzyl-1H-tetrazol-5-yl)-2-chloro-2H-azirine-3-carboxylate* (**15a**): 2-Chloro-2*H*-azirine **15a** was obtained as a yellow oil (97%). IR (film) 701, 723, 1095, 1247, 1408, 1729 cm^−^^1^; ^1^H-NMR (CDCl_3_) δ (ppm) 1.24 (t, 3H, *J* = 6.8 Hz), 4.18–4.24 (m, 2H), 5.90 (d, *J* = 14.8 Hz, 1H), 5.97 (d, *J* = 14.8 Hz, 1H), 7.39 (br s, 5H, Ar-H); ^13^C-NMR (CDCl_3_) δ (ppm) 13.9, 51.9, 53.2, 64.3, 128.4, 129.4, 129.8, 131.9, 140.1, 156.8, 164.9; HRMS (ESI) calcd for C_13_H_13_ClN_5_O_2_ 306.0752 [M + H]^+^, found 306.0752 (Figures S48–S53).

*Ethyl 2-(1-benzyl-1H-tetrazol-5-yl)-2-bromo-2H-azirine-3-carboxylate* (**15b**): 2-Bromo-2*H*-azirine **15b** was obtained as a yellow oil (85%). IR (film) 709, 723, 1013, 1100, 1245, 1445, 1729 cm^−1^; ^1^H-NMR (CDCl_3_) δ (ppm) 1.23 (t, *J* = 6.8 Hz, 3H), 4.16–4.26 (m, 2H), 5.89 (d, *J* = 14.8 Hz, 1H), 5.96 (d, *J* = 14.8 Hz, 1H), 7.16–7.39 (m, 5H, Ar-H); ^13^C-NMR (CDCl_3_) δ (ppm) 13.9, 40.7, 53.2, 64.5, 128.4, 129.4, 129.8, 131.9, 140.2, 157.4, 164.5; HRMS (ESI) calcd for C_13_H_13_BrN_3_O_3_ 350.0247 [M + H]^+^, found 350.0243.

*2-(1-Benzyl-1H-tetrazol-5-yl)-2-chloro-3-phenyl-2H-azirine* (**15c**): 2-Chloro-2*H*-azirine **15c** was obtained as an orange oil (85%). IR (film) 686, 721, 831, 1068, 1451, 1498, 1597, 1743 cm^−1^; ^1^H-NMR (CDCl_3_) δ (ppm) 5.91 (d, *J* = 15.2 Hz, 1H), 5.95 (d, *J* = 15.2 Hz, 1H), 7.37–7.40 (m, Ar-H, 3H), 7.43–7.45 (m, Ar-H, 2H), 7.61–7.65 (m, Ar-H, 2H), 7.73–7.77 (m, Ar-H, 1H), 8.04 (d, *J* = 8.4 Hz, 2H); ^13^C-NMR (CDCl_3_) δ (ppm) 46.4, 52.1, 119.7, 128.5, 129.0, 129.1, 129.8, 131.3, 133.1, 135.7, 152.9, 168.6; HRMS (ESI) calcd for C_16_H_13_ClN_5_ 310.0854 [M + H]^+^, found 310.0854.

*2-(1-Benzyl-1H-tetrazol-5-yl)-2-bromo-3-phenyl-2H-azirine* (**15d**): 2-Bromo-2*H*-azirine **15d** was obtained as an orange oil (87%). IR (film) 685, 720, 808, 1114, 1451, 1498, 1597, 1743 cm^−1^; ^1^H-NMR (CDCl_3_) δ (ppm) 5.91 (d, *J* = 14.8Hz, 1H), 5.96 (d, *J* = 14.8 Hz, 1H), 7.37–7.39 (m, Ar-H, 3H), 7.45–7.47 (m, Ar-H, 2H), 7.62–7.66 (m, Ar-H, 2H), 7.74–7.78 (m, Ar-H, 1H), 8.09 (d, *J* = 8.4 Hz, 2H); ^13^C-NMR (CDCl_3_) δ (ppm) 33.8, 52.1, 119.7, 128.6, 129.0, 129.1, 129.8, 131.4, 133.0, 135.8, 153.2, 169.6; HRMS (ESI) calcd for C_16_H_13_BrN_5_ 354.0348 [M + H]^+^, found 354.0343.

*2-(1-Benzyl-1H-tetrazol-5-yl)-2-chloro-3-(thiophen-2-yl)-2H-azirine* (**15e**): 2-Chloro-2*H*-azirine **15e** was obtained as a brown oil (99%). IR (film) 699, 720, 805, 1033, 1408, 1498, 1742 cm^−1^; ^1^H-NMR (CDCl_3_) δ (ppm) 5.91 (s, 2H), 7.35–7.44 (m, Ar-H, 6H), 7.99 (d, *J* = 3.2 Hz, 1H), 8.06 (d, *J* = 4.8 Hz); ^13^C-NMR (CDCl_3_) δ (ppm) 47.0, 52.2, 121.1, 128.5, 129.1, 129.4, 133.1, 138.3, 138.9, 152.7, 161.7; HRMS (ESI) calcd for C_14_H_11_ClN_5_S 316.0418 [M + H]^+^, found 316.0417.

*2-(1-Benzyl-1H-tetrazol-5-yl)-2-bromo-3-(thiophen-2-yl)-2H-azirine* (**15f**): 2-Bromo-2*H*-azirine **15f** was obtained as a brown oil (99%). IR (film) 698, 719, 790, 856, 1030, 1406, 1497, 1739 cm^−1^; ^1^H-NMR (CDCl_3_) δ (ppm) 5.90 (d, *J* = 14.8Hz, 1H), 5.95 (d, *J* = 14.8 Hz, 1H), 7.37–7.41 (m, Ar-H, 4H), 7.44–7.47 (m, Ar-H, 2H), 8.08–8.10 (m, 2H); ^13^C-NMR (CDCl_3_) δ (ppm) 34.5, 52.1, 121.2, 128.6, 129.1, 129.5, 133.0, 138.6, 139.2, 153.0, 162.7; HRMS (ESI) calcd for C_14_H_11_BrN_5_S 359.9913 [M + H]^+^, found 359.9912.

*2-(1-Benzyl-1H-tetrazol-5-yl)-2-chloro-3-(furan-2-yl)-2H-azirine* (**15g**): 2-Chloro-2*H*-azirine **15g** was obtained as an oil (98%). IR (film) 699, 719, 883, 1016, 1072, 1455, 1747 cm^−1^; ^1^H-NMR (CDCl_3_) δ (ppm) 5.90 (s, 2H), 6.78 (dd, *J* = 1.2 Hz and *J* = 3.6 Hz, 1H), 7.37–7.43 (m, Ar-H, 5H), 7.56 (d, *J* = 3.6 Hz, 1H), 7.97 (d, *J* = 1.2 Hz, 1H); ^13^C-NMR (CDCl_3_) δ (ppm) 46.1, 52.1, 113.9, 125.6, 128.5, 129.1, 133.0, 136.9, 151.7, 152.5, 157.7; HRMS (ESI) calcd for C_14_H_11_ClN_5_O 300.0647 [M + H]^+^, found 300.0648.

*2-(1-Benzyl-1H-tetrazol-5-yl)-2-bromo-3-(furan-2-yl)-2H-azirine* (**15h**): 2-Bromo-2*H*-azirine **15h** was obtained as a brown oil (99%). IR (film) 699, 719, 770, 1016, 1072, 1454, 1498, 1747 cm^−1^; ^1^H-NMR (CDCl_3_) δ (ppm) 5.89 (d, *J* = 15.2 Hz, 1H), 5.93 (d, *J* = 15.2 Hz, 1H), 6.80 (d, *J* = 2.0 Hz, 1H), 7.37–7.40 (m, Ar-H, 3H), 7.44–7.45 (m, Ar-H, 2H), 7.63 (d, *J* = 3.6 Hz, 1H), 8.00 (br s, 1H); ^13^C-NMR (CDCl_3_) δ (ppm) 33.3, 52.1, 114.0, 126.0, 128.6, 129.0, 132.9, 137.0, 152.0, 152.8, 158.5; HRMS (ESI) calcd for C_14_H_11_BrN_5_O 344.0142 [M + H]^+^, found 344.0143.

#### 3.2.5. X-ray Crystallography Structure Determination

X-ray data for compounds **7c**, **7h** and **7e** were collected on a Bruker APEXII diffractometer (Mo Kα radiation, graphite monochromator, λ = 0.71073 Å) using φ and ω scans. Data integration and scaling were performed with the SAINT suite of programs [35] and SADABS [35] was used for an empirical absorption collection based on a measurement of a large set of redundant reflections. All structures were solved by direct methods using SHELXT-2014/7 [36] and full-matrix least squares refinement of the structural model was performed by SHELXL-2014/7 [37]. All non-H atoms were refined anisotropically. H atoms were placed at calculated idealized positions and refined as riding using SHELXL-2014/7 default values. A summary of the data collection and refinement details is given in Table 1. Crystallographic figures and tables were produced using PLATON [38]. CCDC 1432372 (**7c**), CCDC 1432373 (**7h**) and CCDC 1432374 (**7e**) contains the supplementary crystallographic data for this paper. These data can be obtained free of charge via http://www.ccdc.cam.ac.uk/conts/retrieving.html (or from the CCDC, 12 Union Road, Cambridge CB2 1EZ, UK; Fax: + 44 1223 336033; E-mail: deposit@ccdc.cam.ac.uk).

## 4. Conclusions

The selective synthesis of (*Z*)-haloazidoalkenes bearing a tetrazol-5-yl substituent via a non-classical Wittig reaction is reported. The stereochemistry assignment was supported by X-ray crystallography studies. Haloazidoalkenes underwent thermolysis to efficiently give novel 2-halo-2-(tetrazol-5-yl)-2*H*-azirines bearing phenyl, furan-2-yl, thiophen-2-yl and carboxylate substituents at C-3. These 2*H*-azirines can be very useful building blocks for the synthesis of new 5-substituted tetrazoles.

## Supplementary Materials

Supplementary materials can be accessed at: http://www.mdpi.com/1420-3049/20/12/19848/s1.
