# Supplementary material for: Synthesis of New 2-Halo-2-(1H-tetrazol-5-yl)-2H-azirines via a Non-Classical Wittig Reaction
Source: Molecules. 2015 Dec 14;20(12):22351–63. doi: 10.3390/molecules201219848 (PMC6332326; doi:10.3390/molecules201219848)
Supplement: Supplementary file 1 [file molecules-20-19848-s001.pdf]

Supplementary Information

Table of Contents

1. ^{1}H-NMR and ^{13}C-NMR Spectra of new compounds S1
2. X-ray Crystallography Information S24
3. ^{1}H-NMR Experiments of 2*H*-Azirine 15a S24

1. NMR Spectra of New Compounds

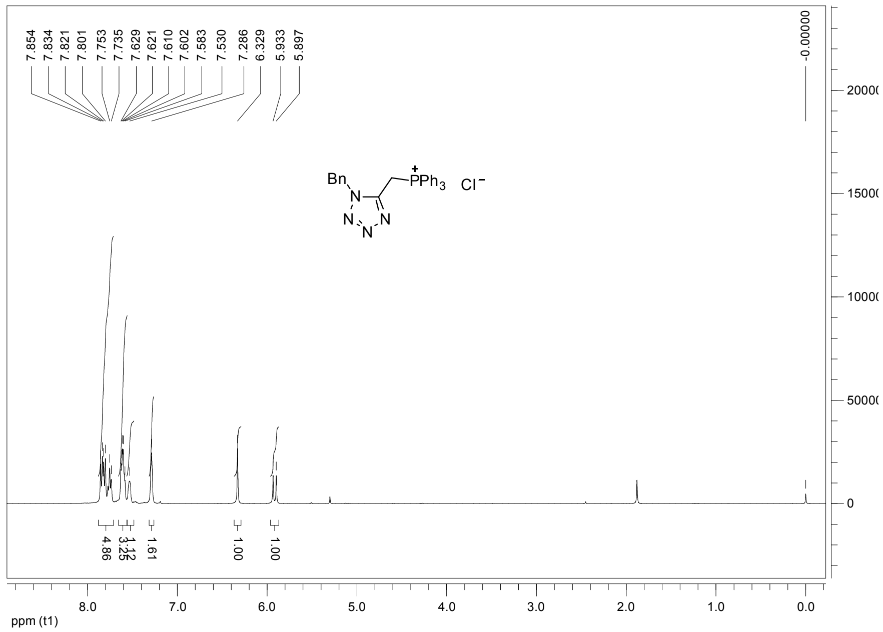

Figure S1. ^{1}H-NMR spectrum of compound **4**.

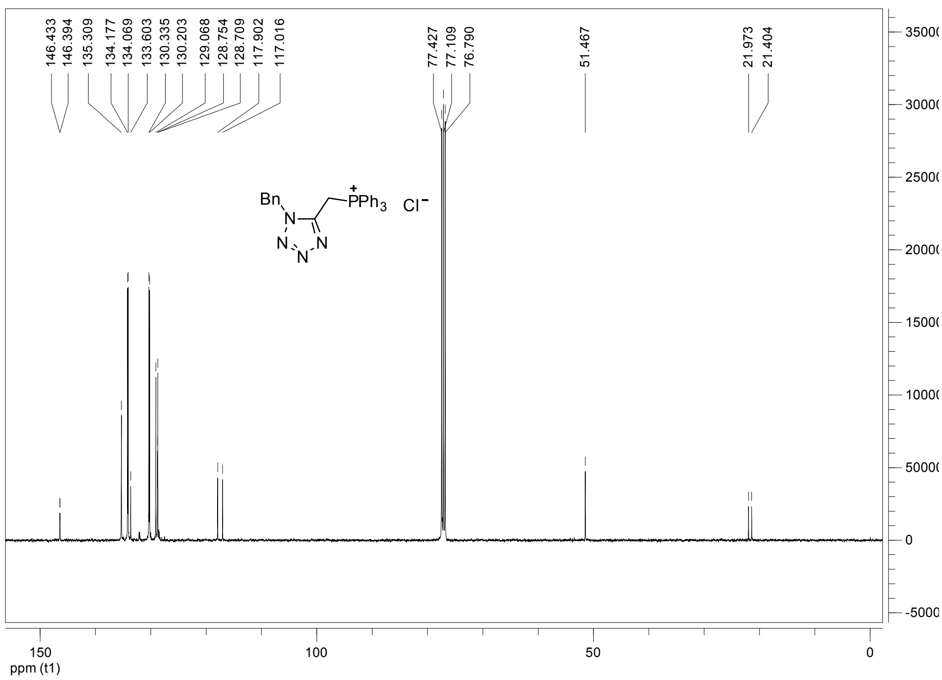

Figure S2. ^{13}C-NMR spectrum of compound **4**.

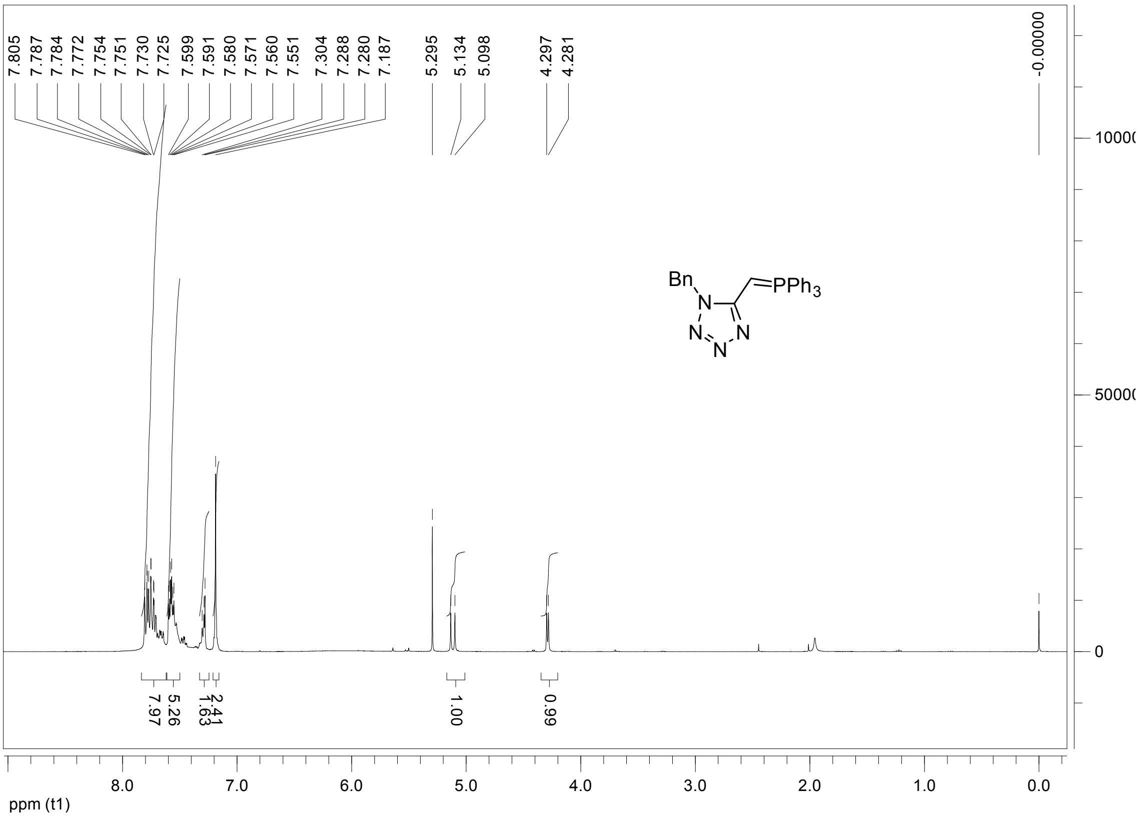

Figure S3. ^{1}H-NMR spectrum of compound **5**.

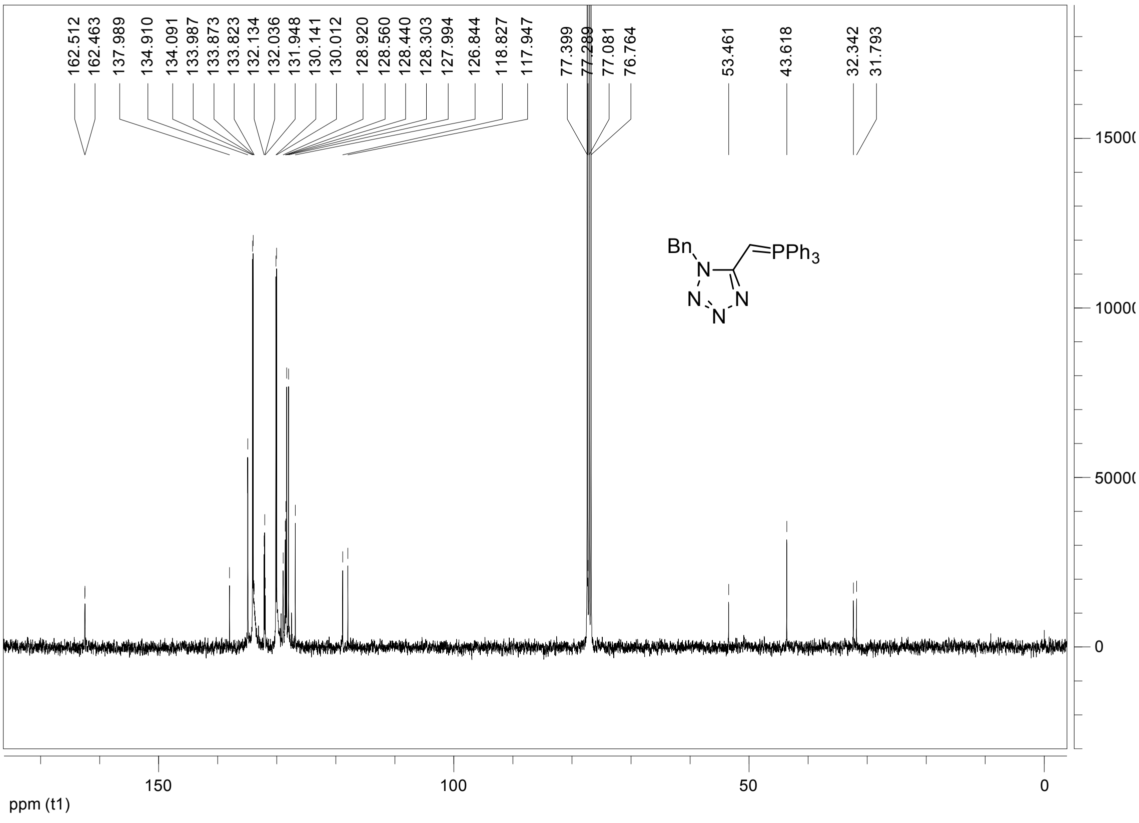

Figure S4. ^{13}C-NMR spectrum of compound **5**.

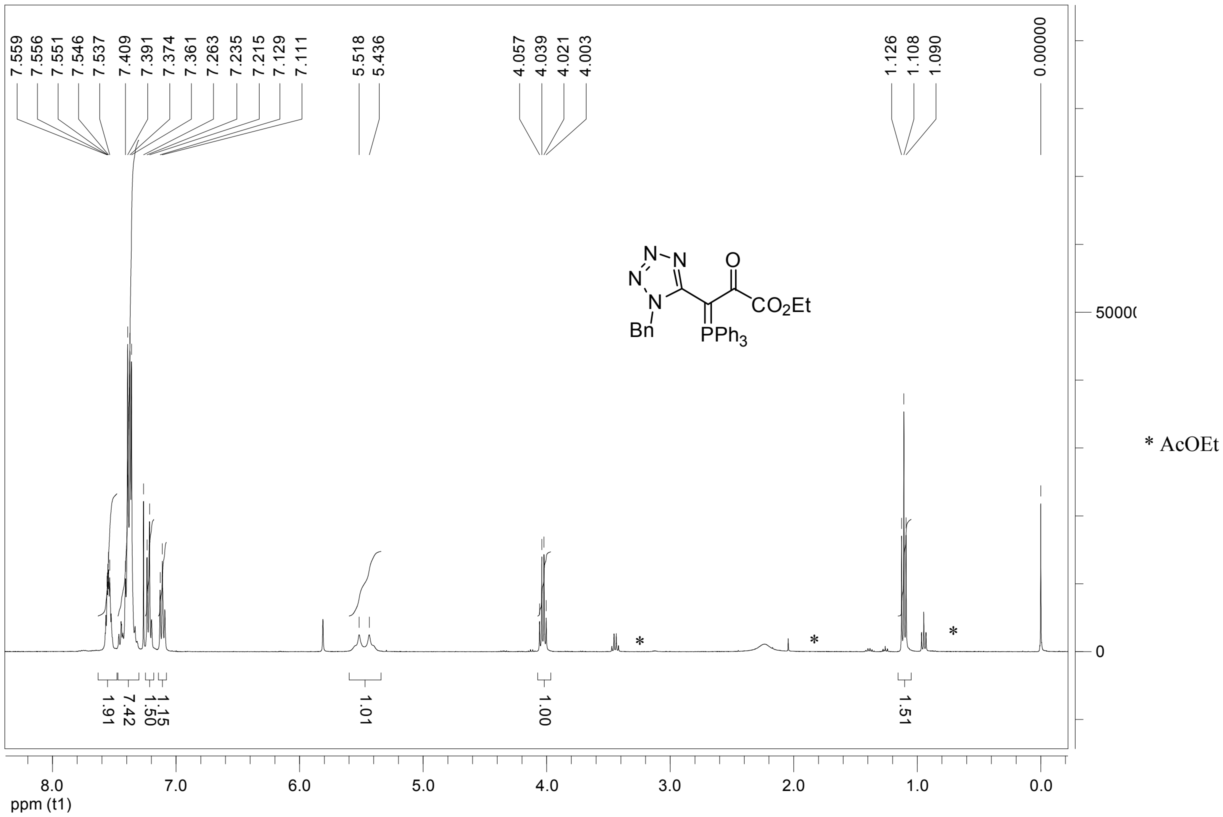

Figure S5. ^{1}H-NMR spectrum of compound **6a**.

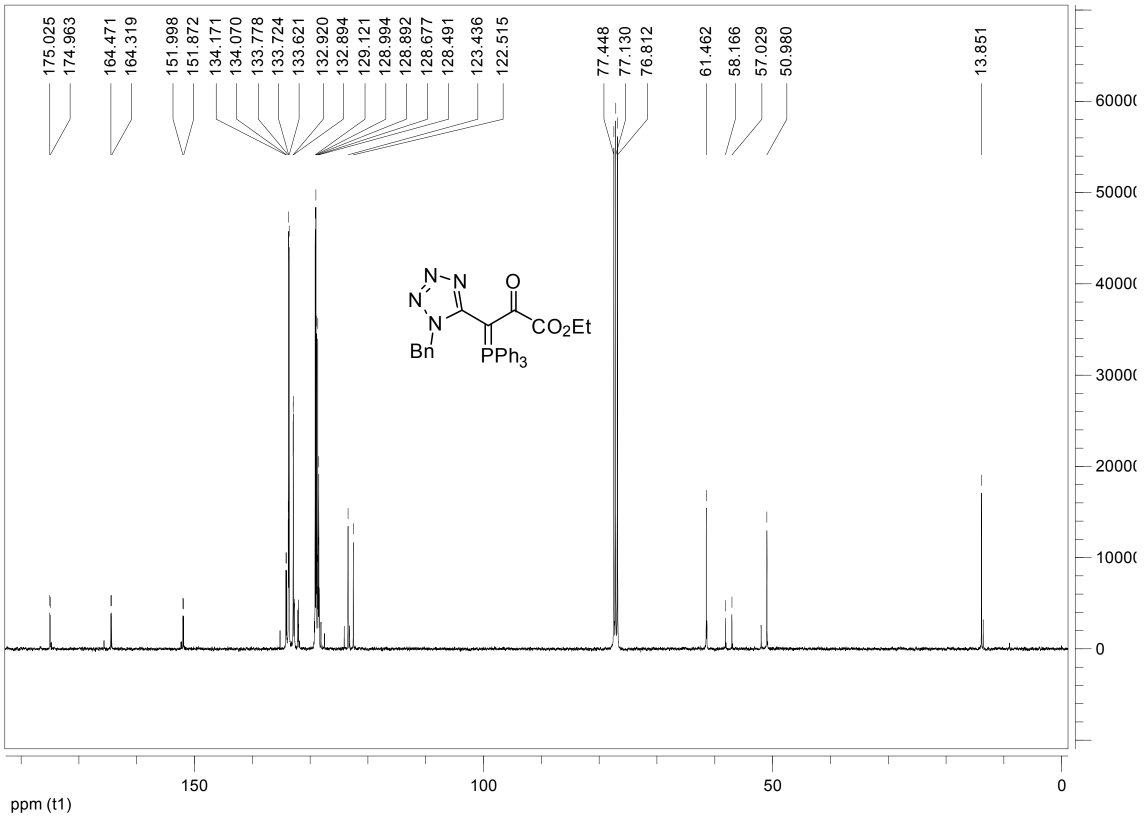

Figure S6. ^{13}C-NMR spectrum of compound **6a**.

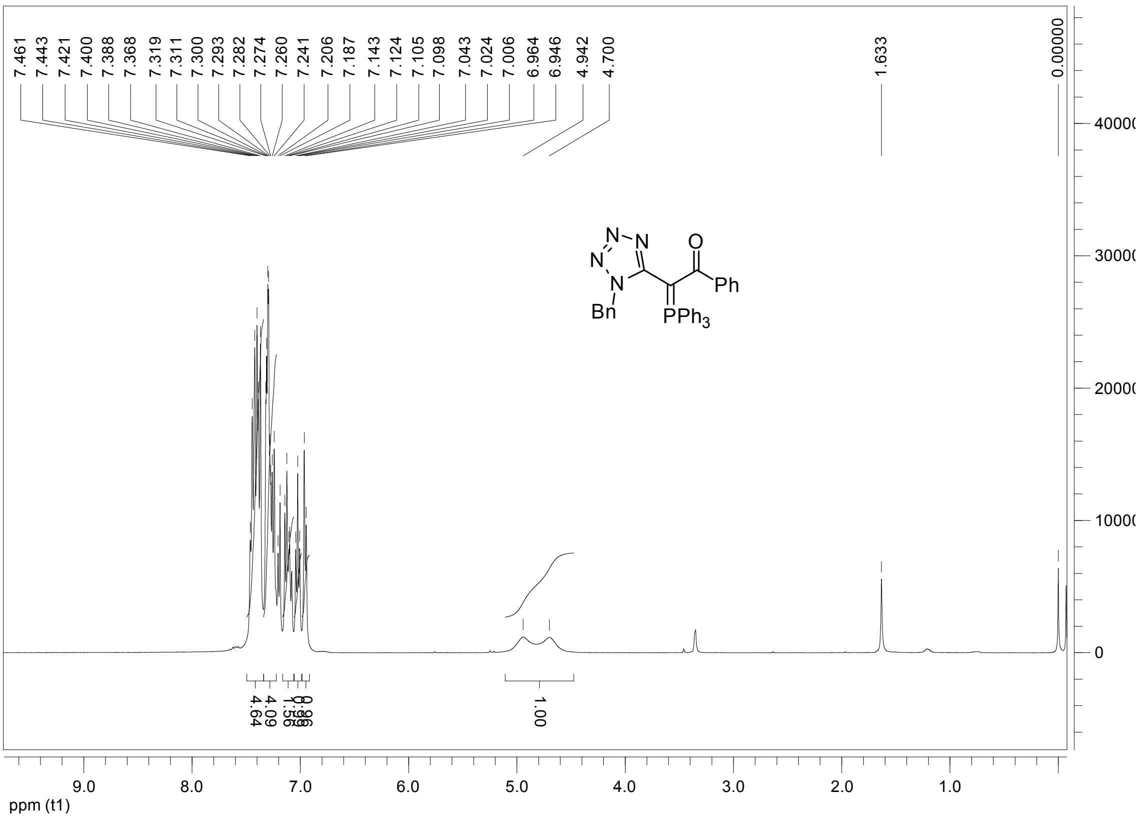

Figure S7. ^{1}H-NMR spectrum of compound **6b**.

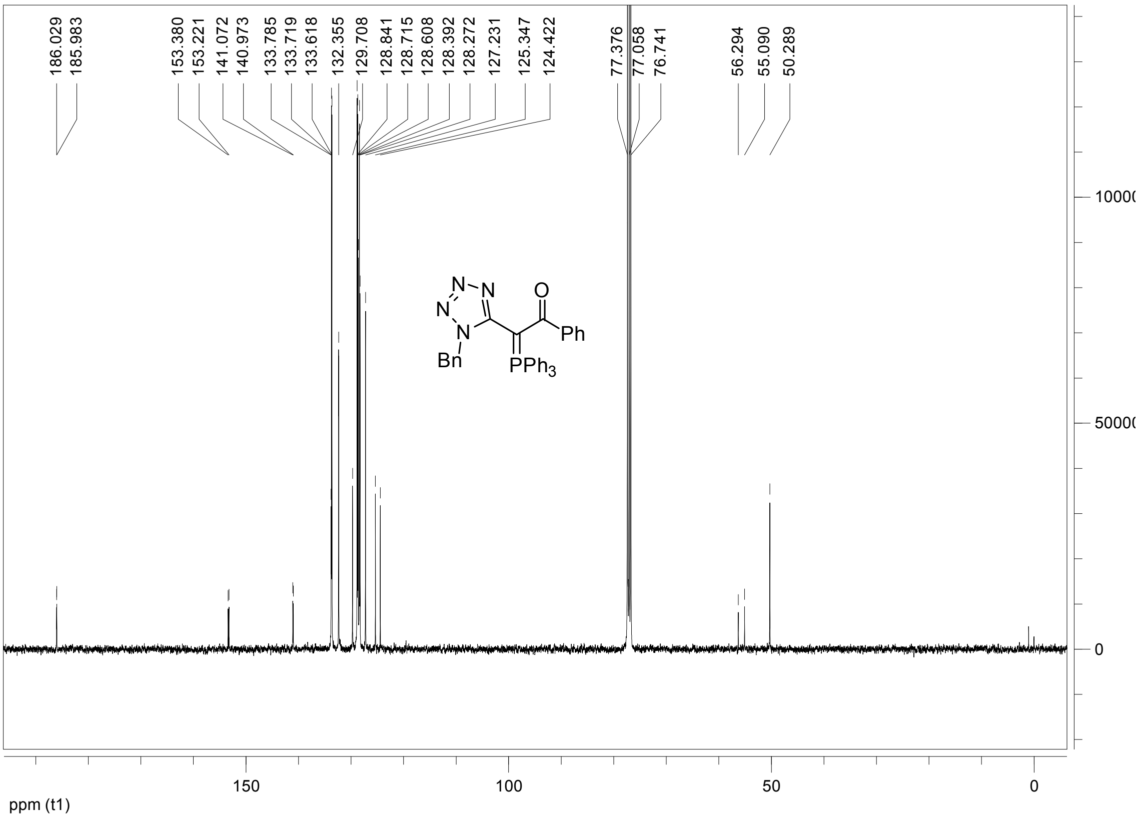

Figure S8. ^{13}C-NMR spectrum of compound **6b**.

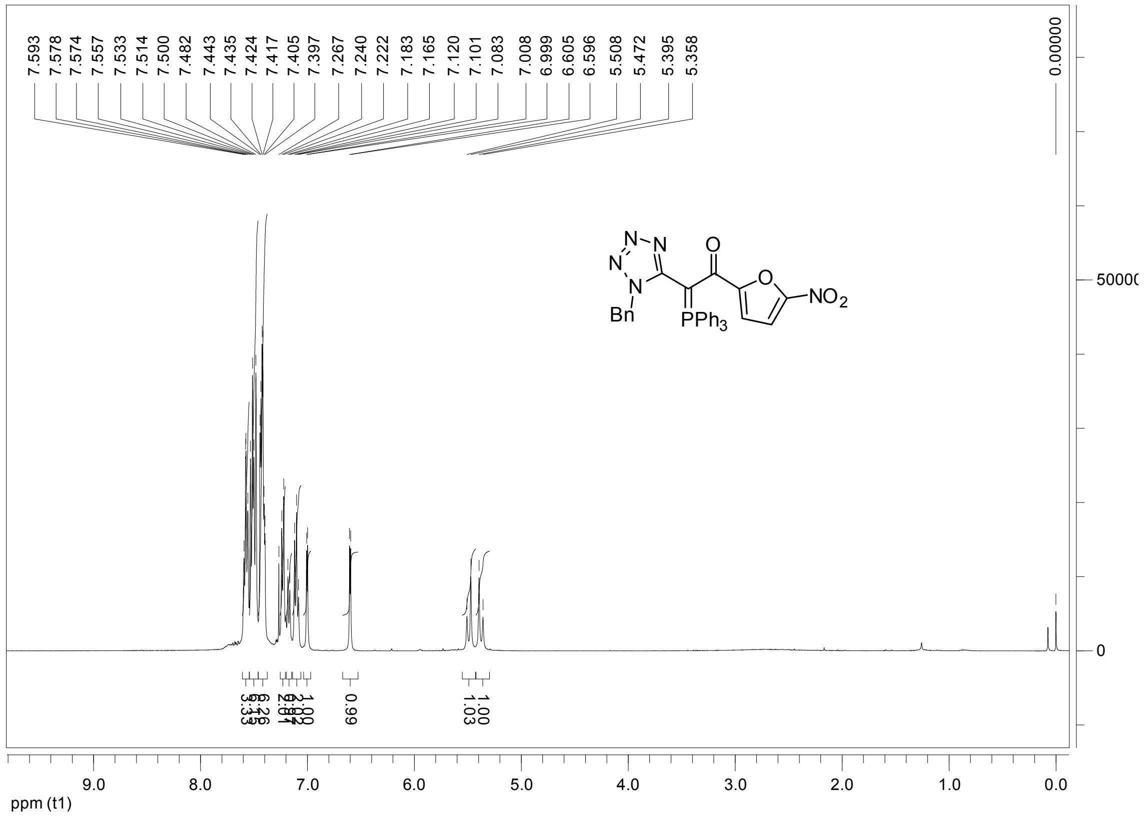

Figure S9. ^{1}H-NMR spectrum of compound **6c**.

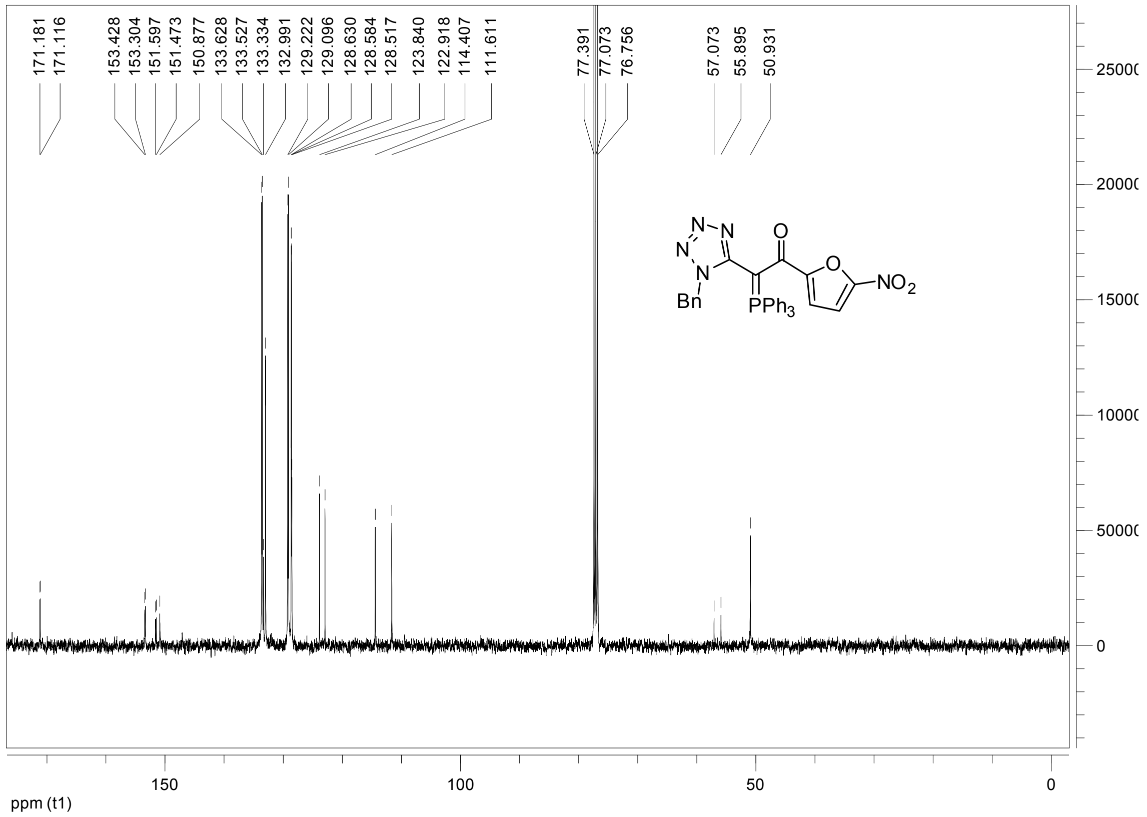

Figure S10. ^{13}C-NMR spectrum of compound **6c**.

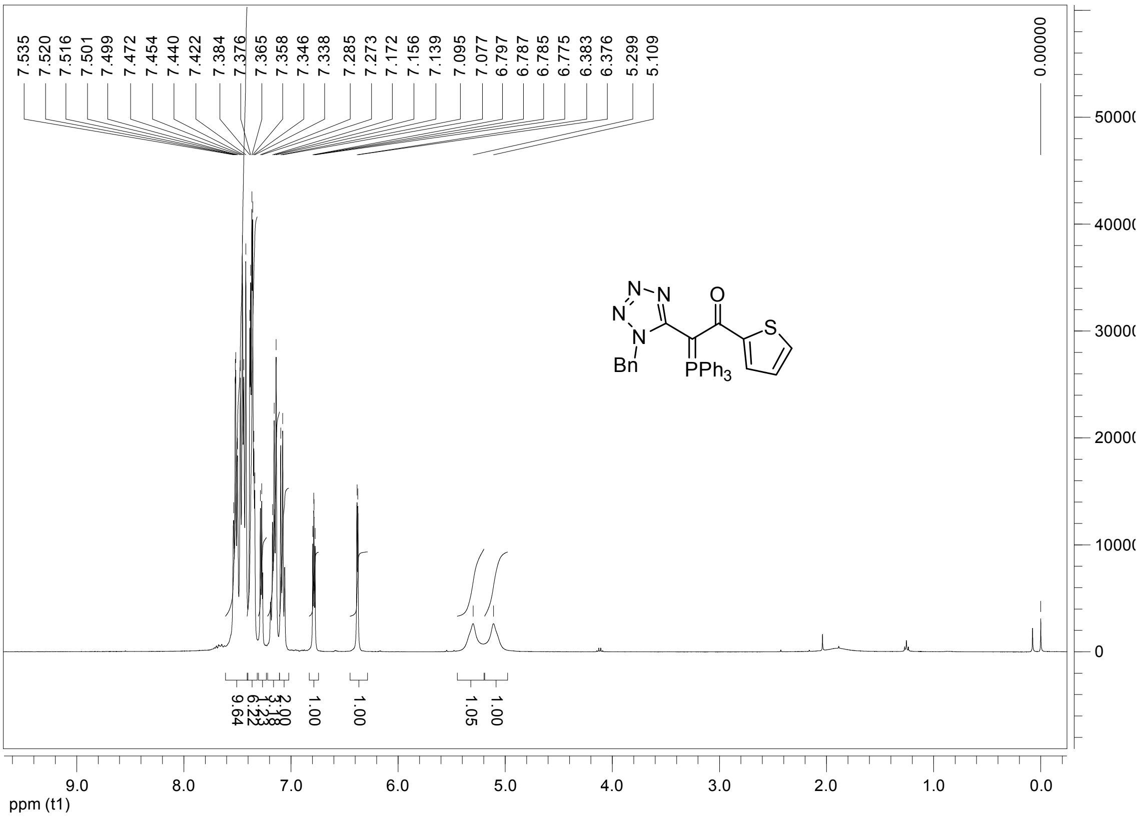

Figure S11. ^{1}H-NMR spectrum of compound **6d**.

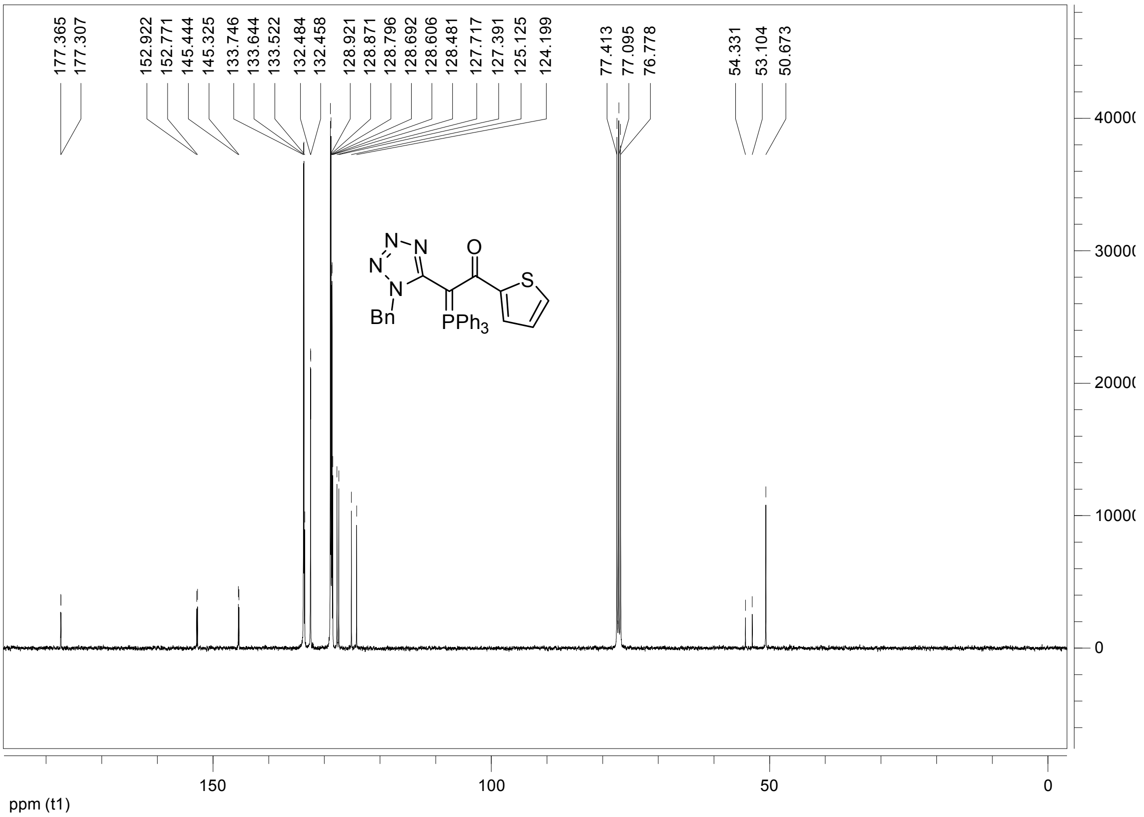

Figure S12. ^{13}C-NMR spectrum of compound **6d**.

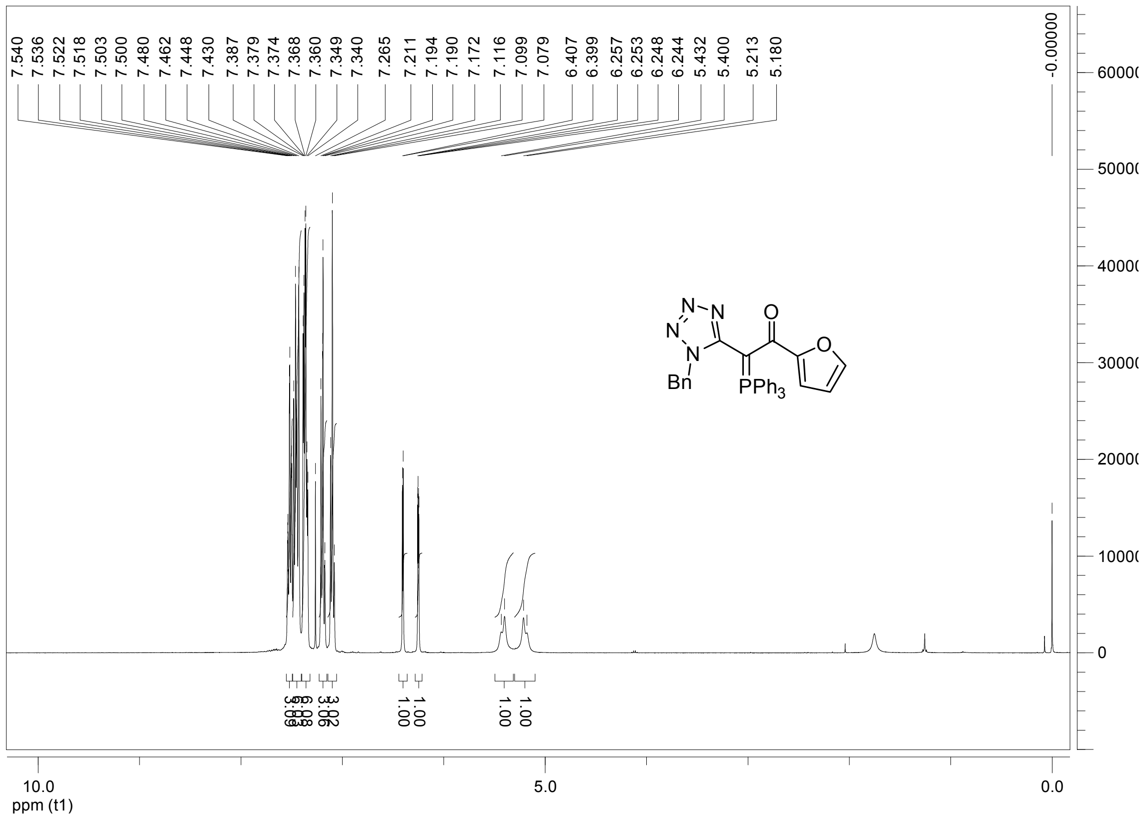

Figure S13. ^{1}H-NMR spectrum of compound **6e**.

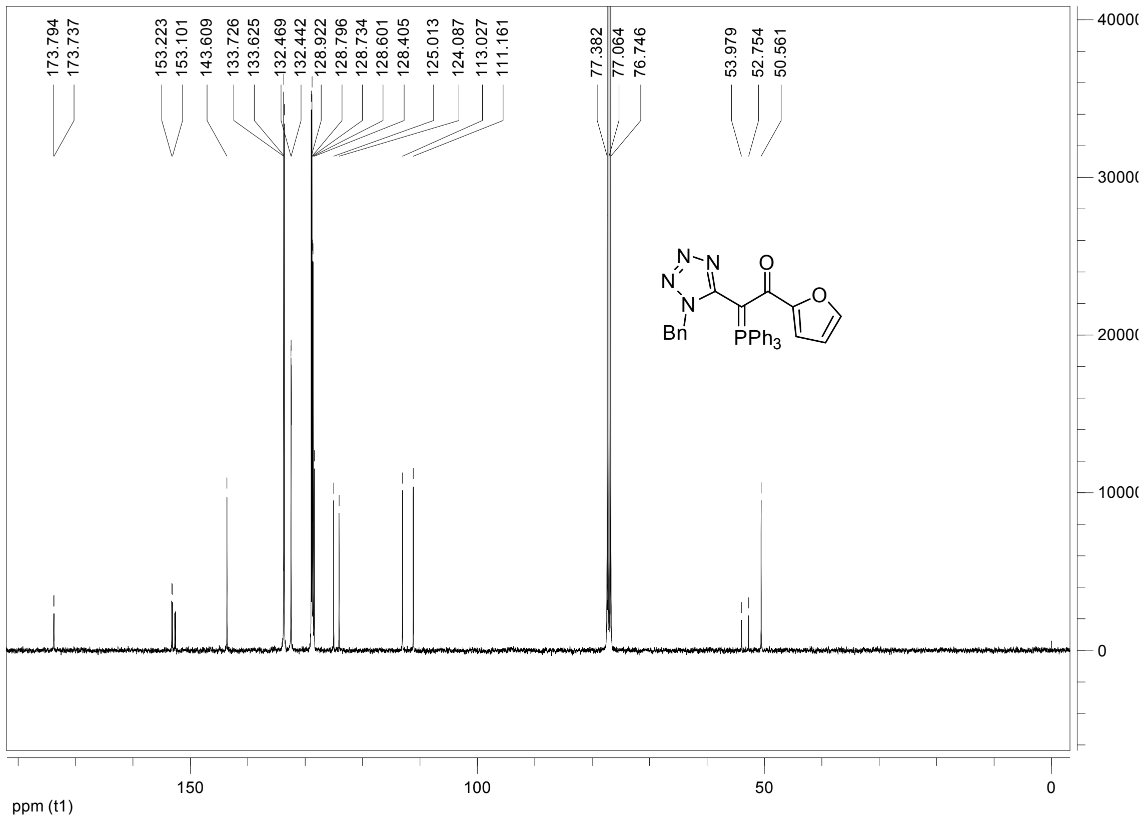

Figure S14. ^{13}C-NMR spectrum of compound **6e**.

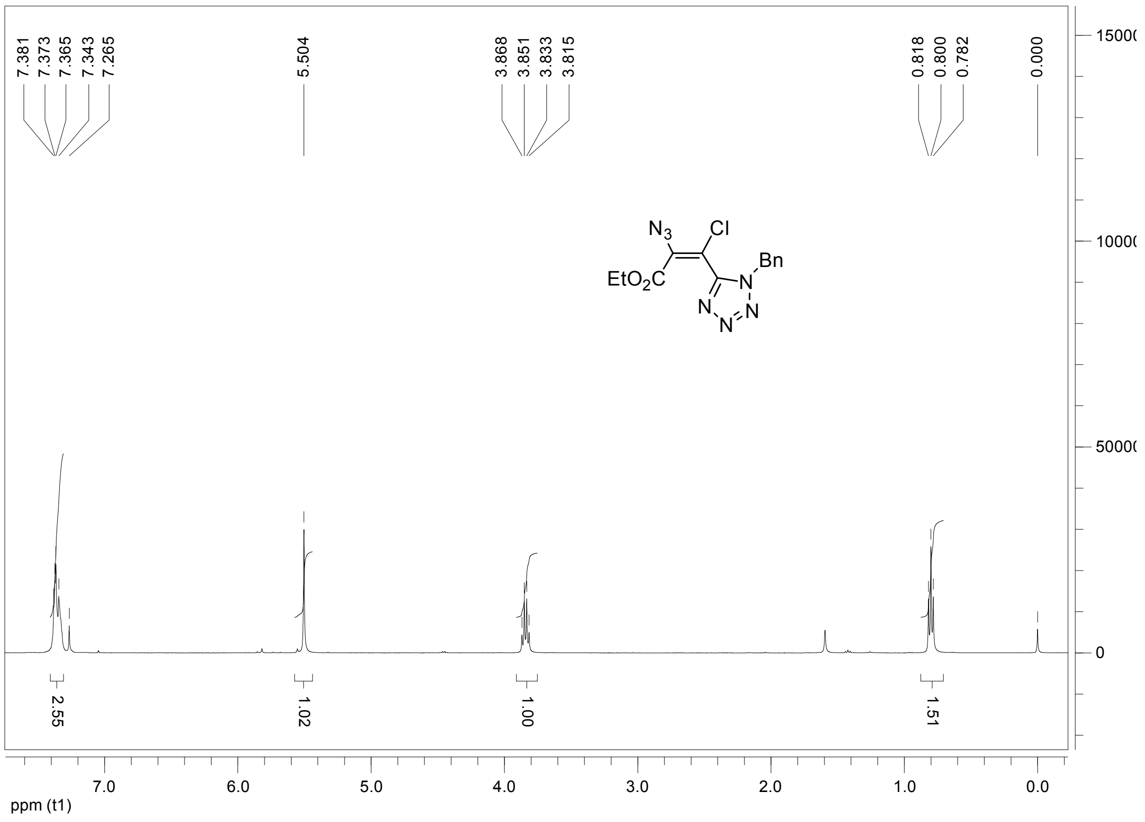

Figure S15. ^{1}H-NMR spectrum of compound **7a**.

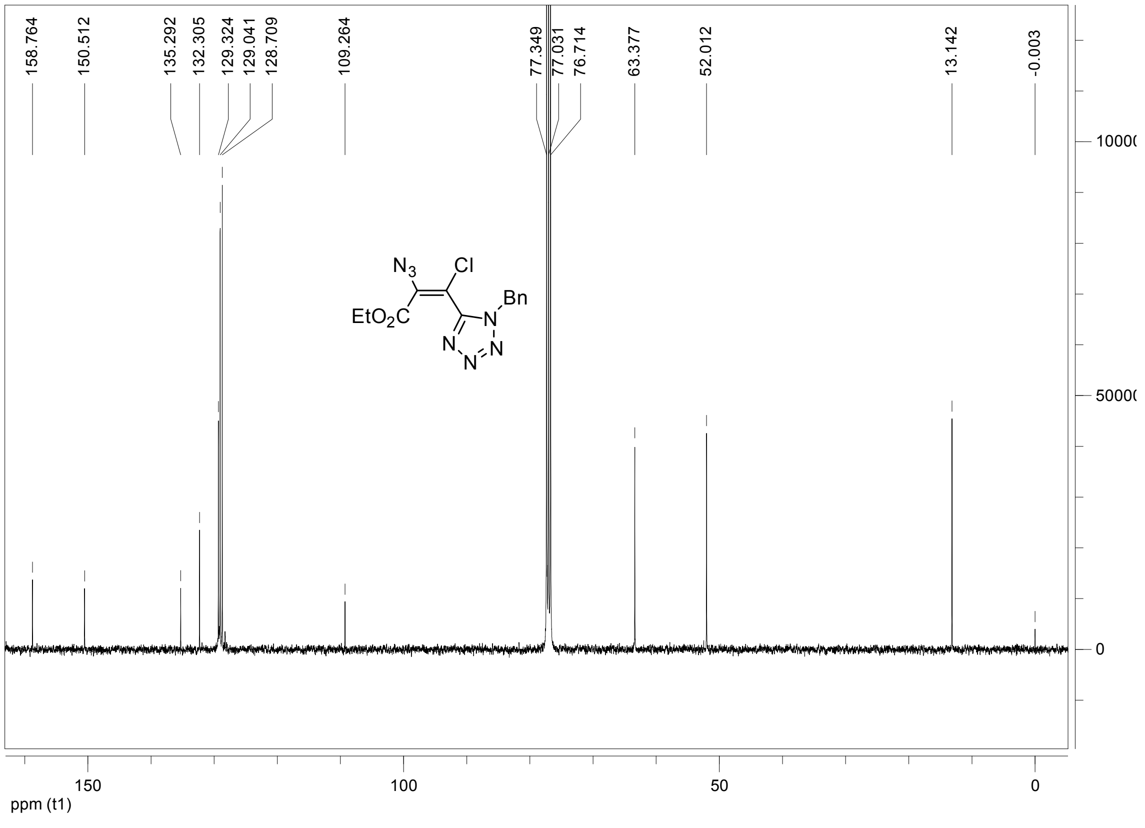

Figure S16. ^{13}C-NMR spectrum of compound **7a**.

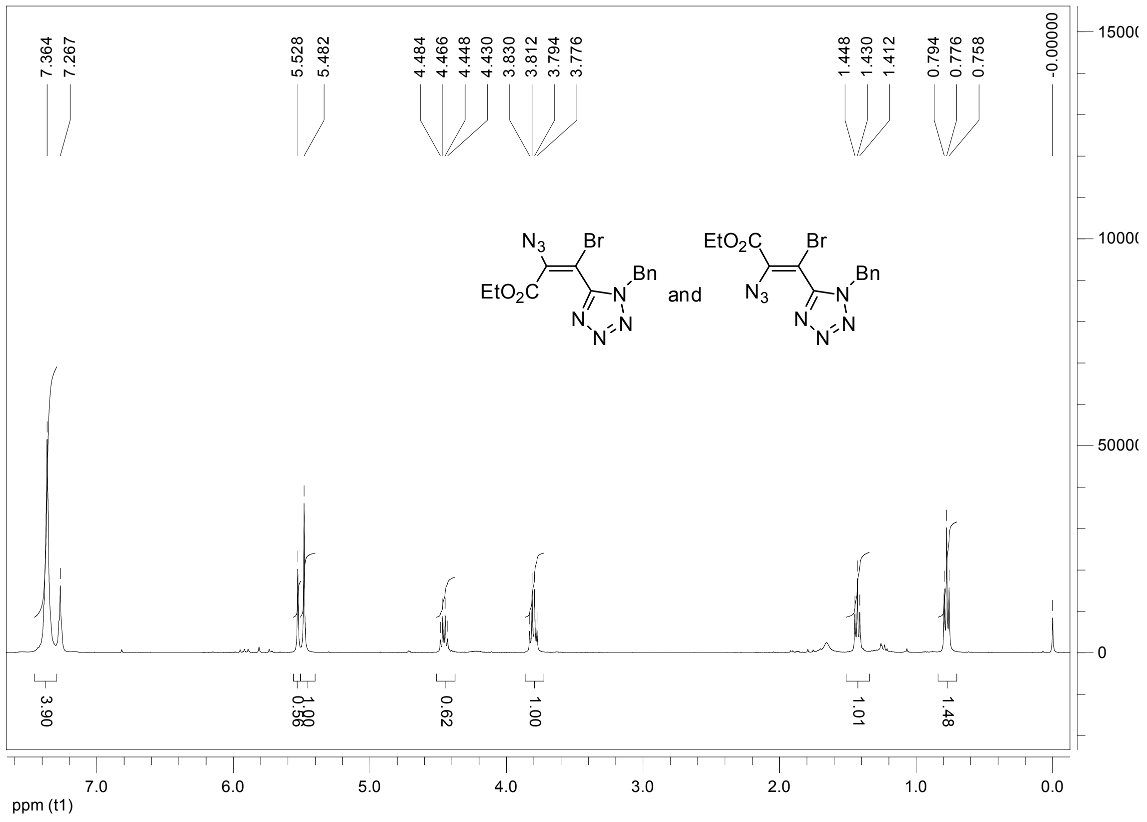

Figure S17. ^{1}H-NMR spectrum of mixture of compounds **7b** and **b**.

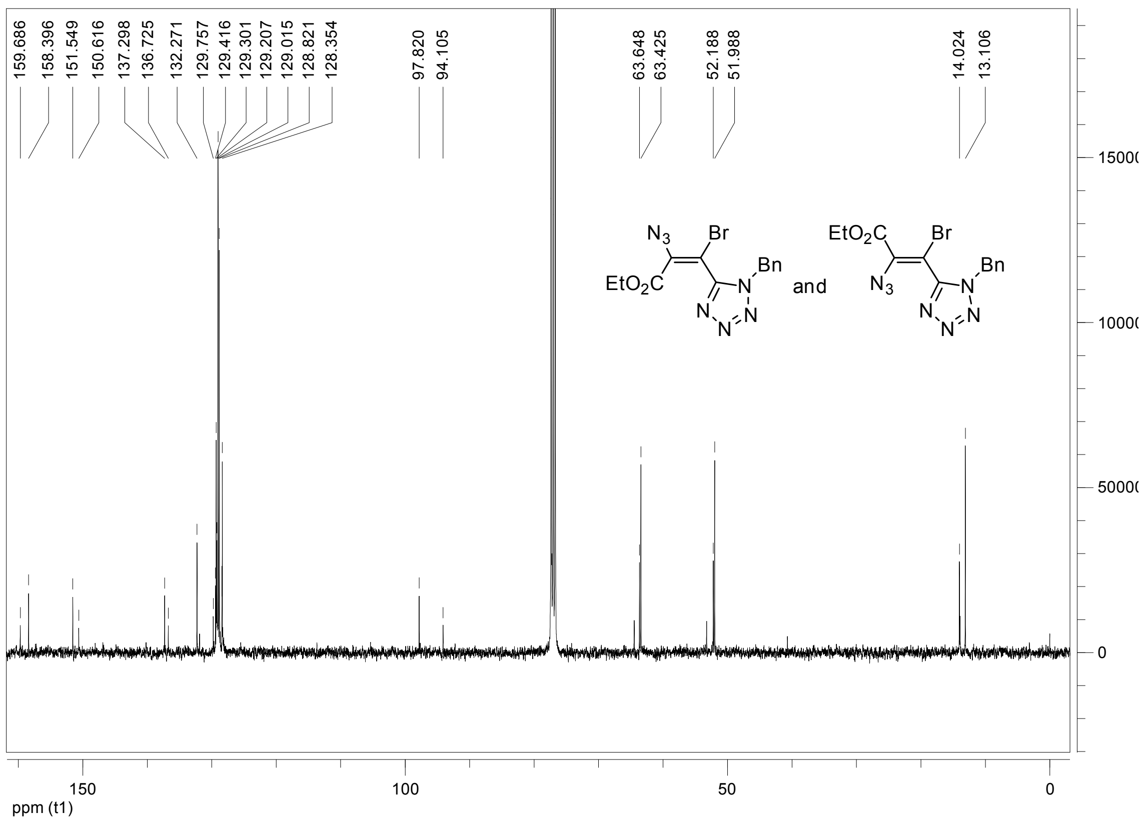

Figure S18. ^{13}C-NMR spectrum of compounds **7b** and **b**.

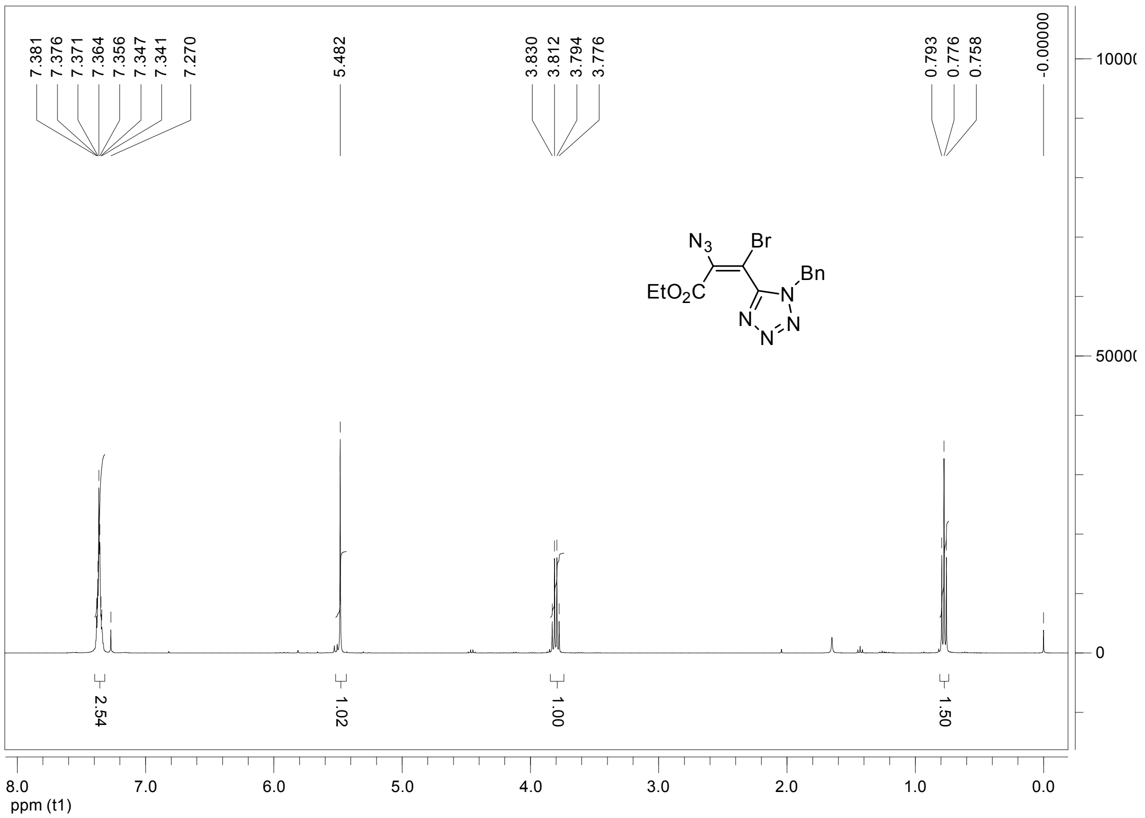

Figure S19. ^{1}H-NMR spectrum of compound **7b**.

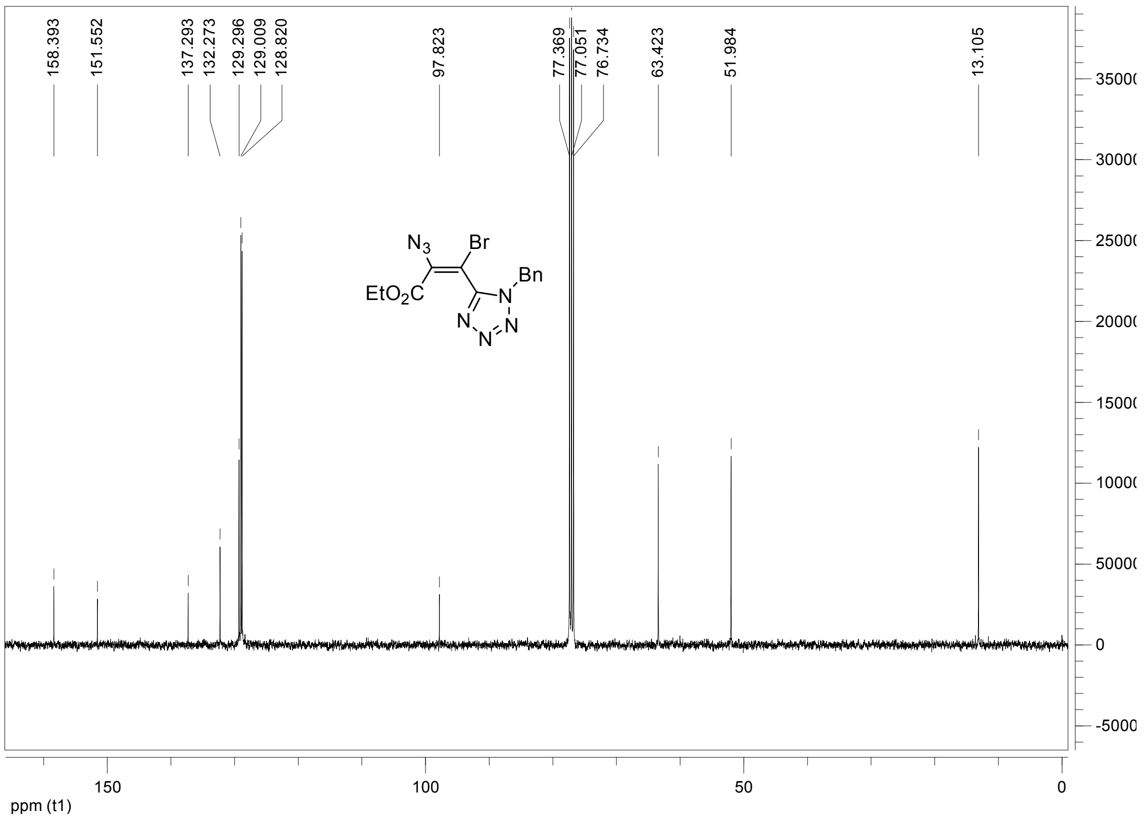

Figure S20. ^{13}C-NMR spectrum of compound **7b**.

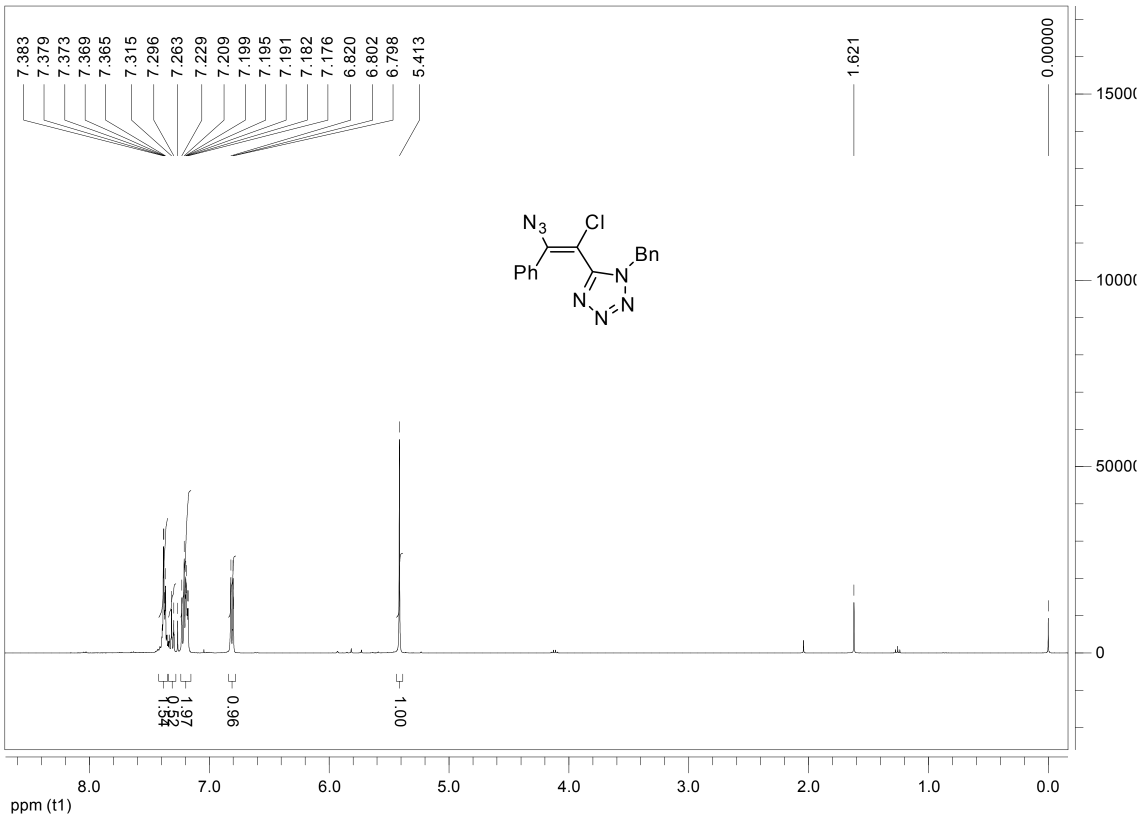

Figure S21. ^{1}H-NMR spectrum of compound **7c**.

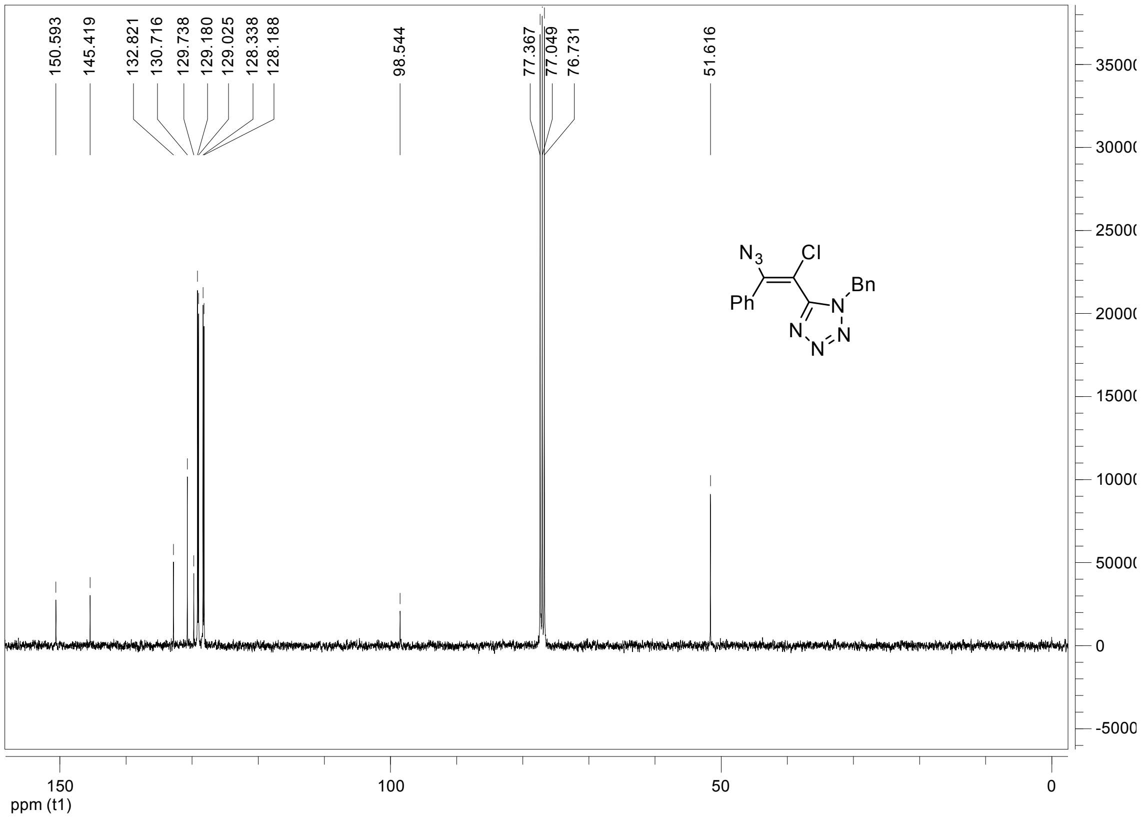

Figure S22. ^{13}C-NMR spectrum of compound **7c**.

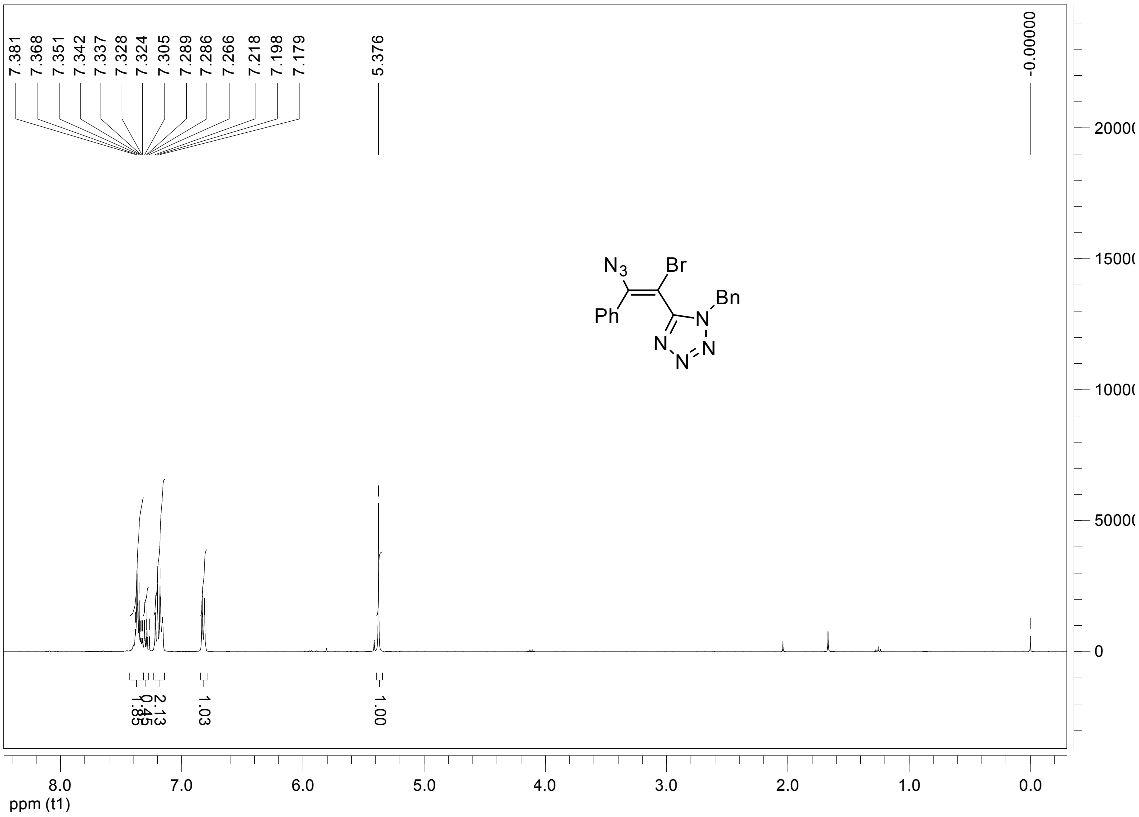

Figure S23. ^{1}H-NMR spectrum of compound **7d**.

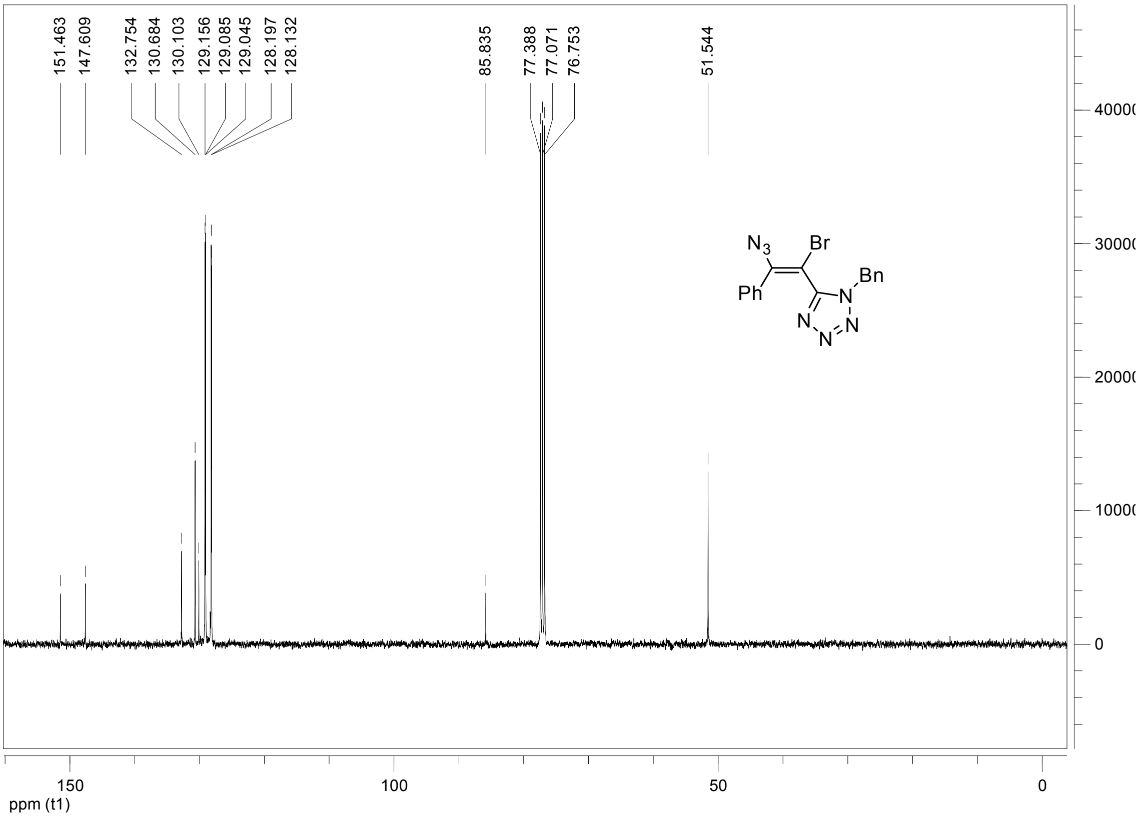

Figure S24. ^{13}C-NMR spectrum of compound **7d**.

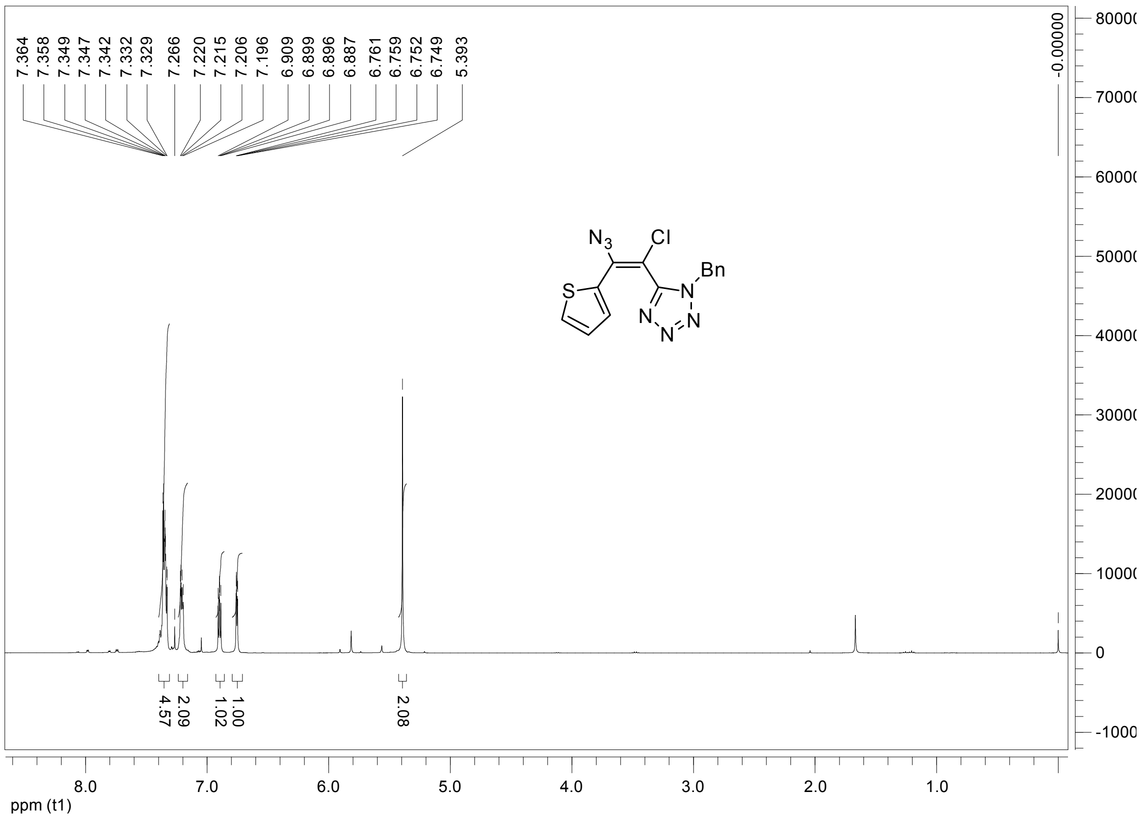

Figure S25. ^{1}H-NMR spectrum of compound **7e**.

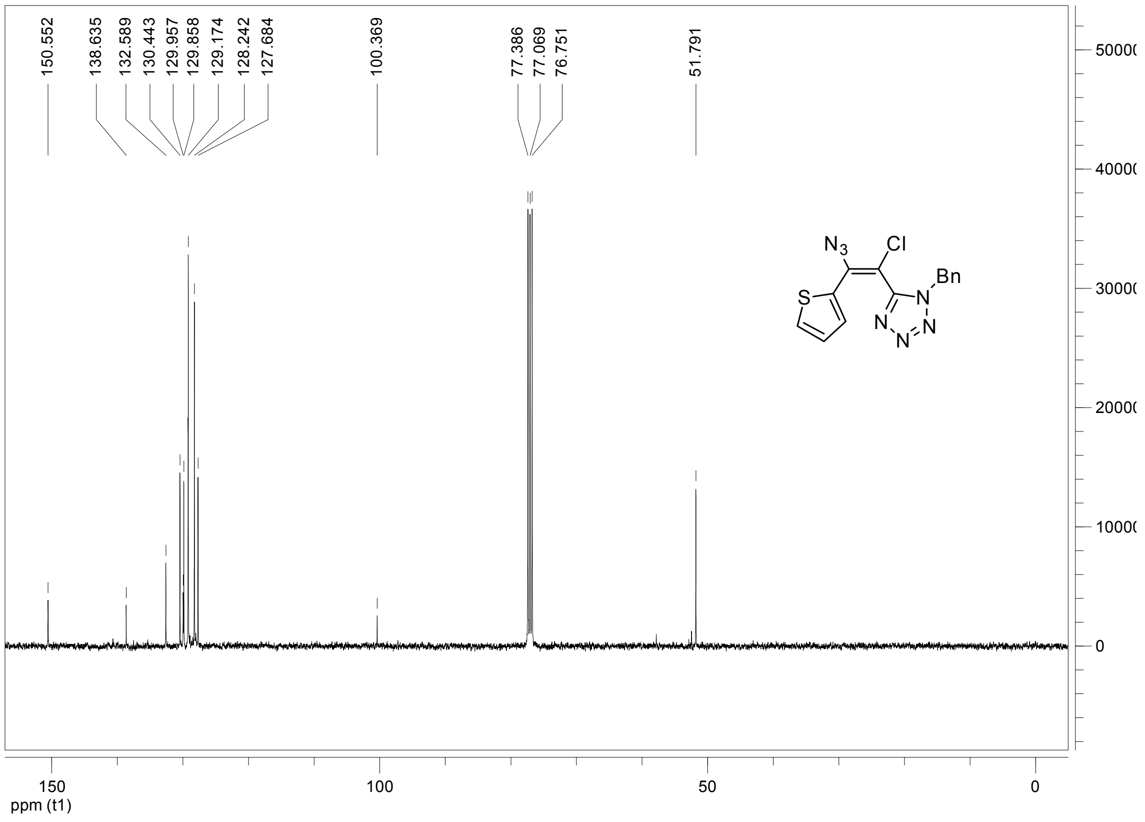

Figure S26. ^{13}C-NMR spectrum of compound **7e**.

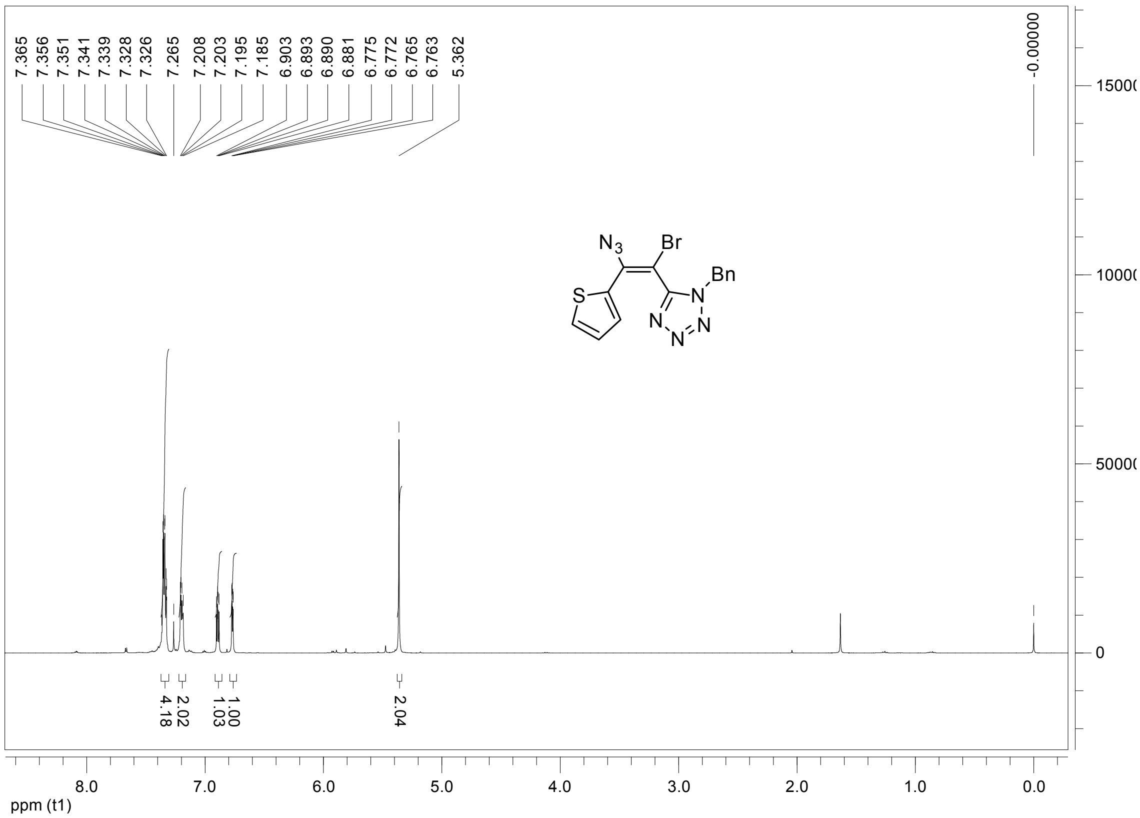

Figure S27. ¹H-NMR spectrum of compound **7f**.

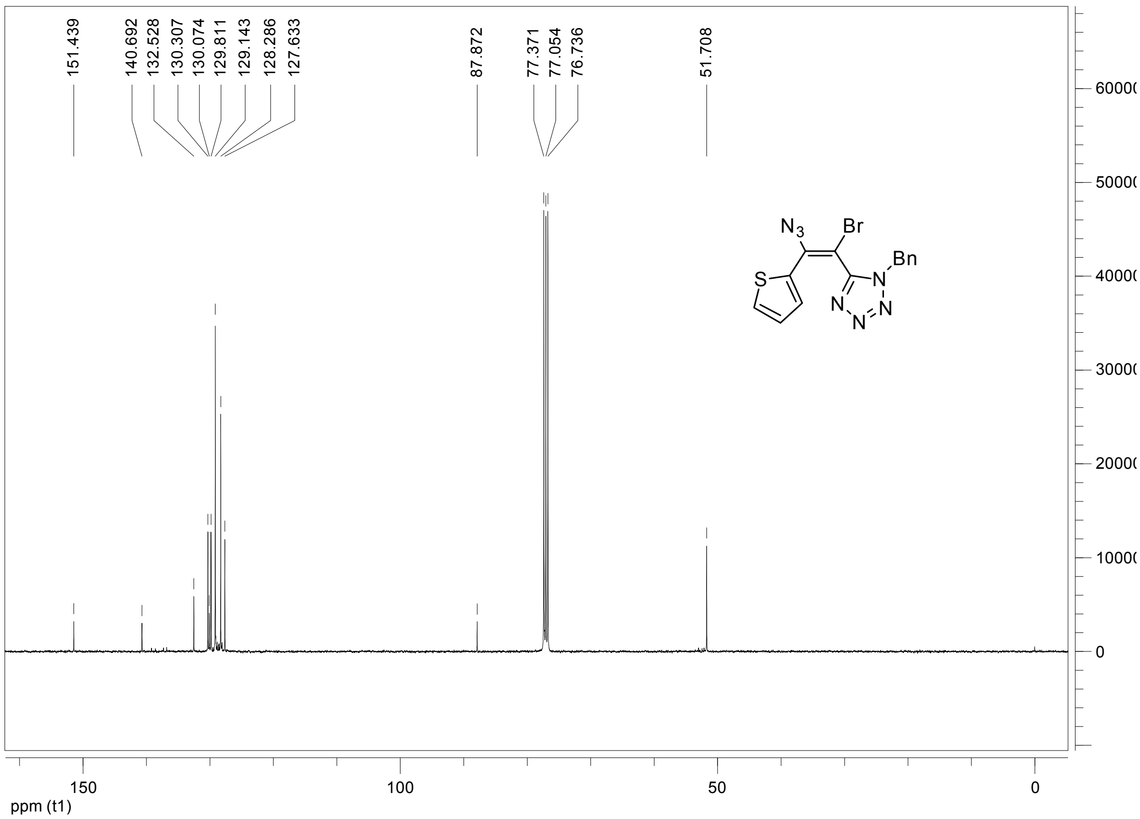

Figure S28. ¹³C-NMR spectrum of compound **7f**.

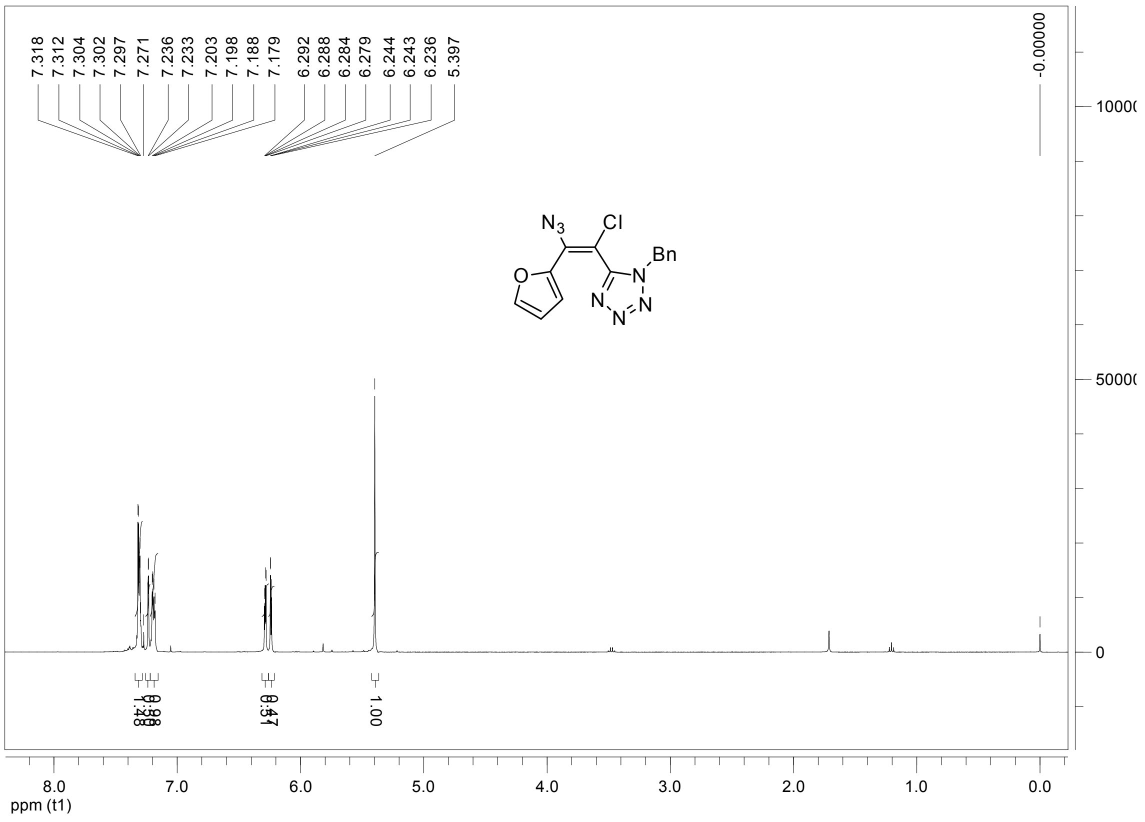

Figure S29. ^{1}H-NMR spectrum of compound **7g**.

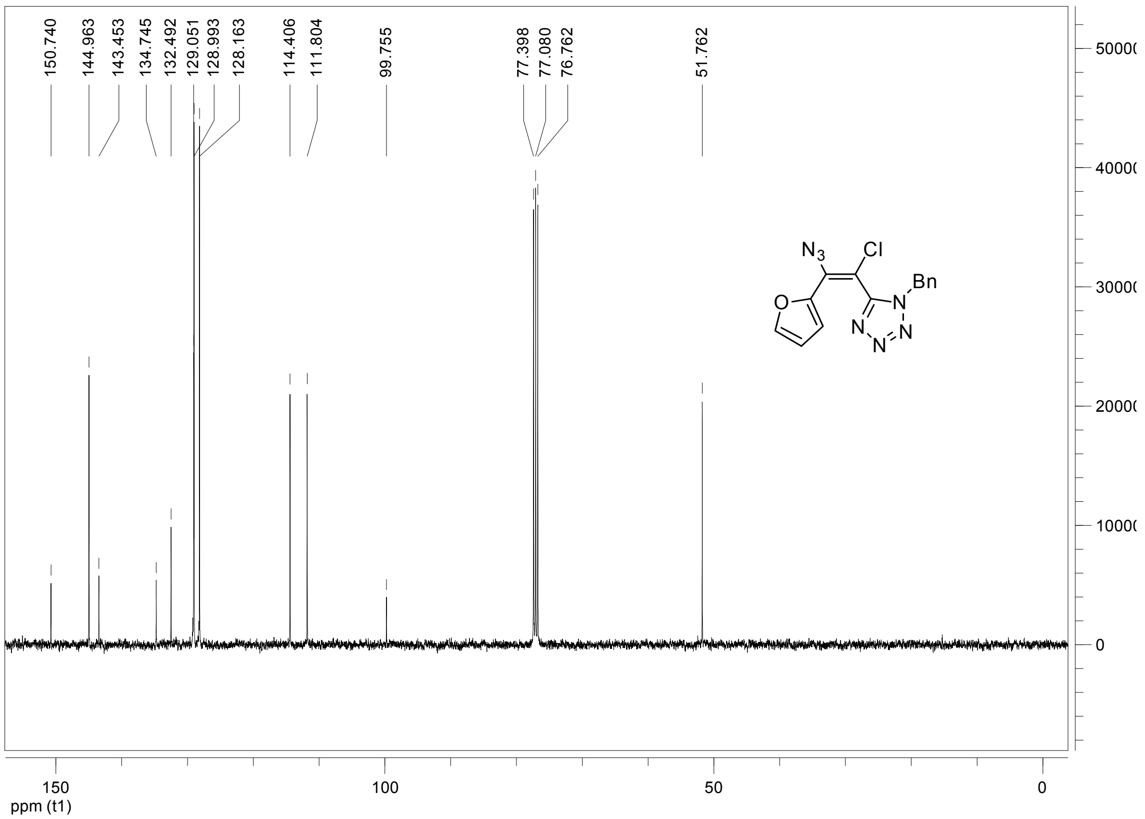

Figure S30. ^{13}C-NMR spectrum of compound **7g**.

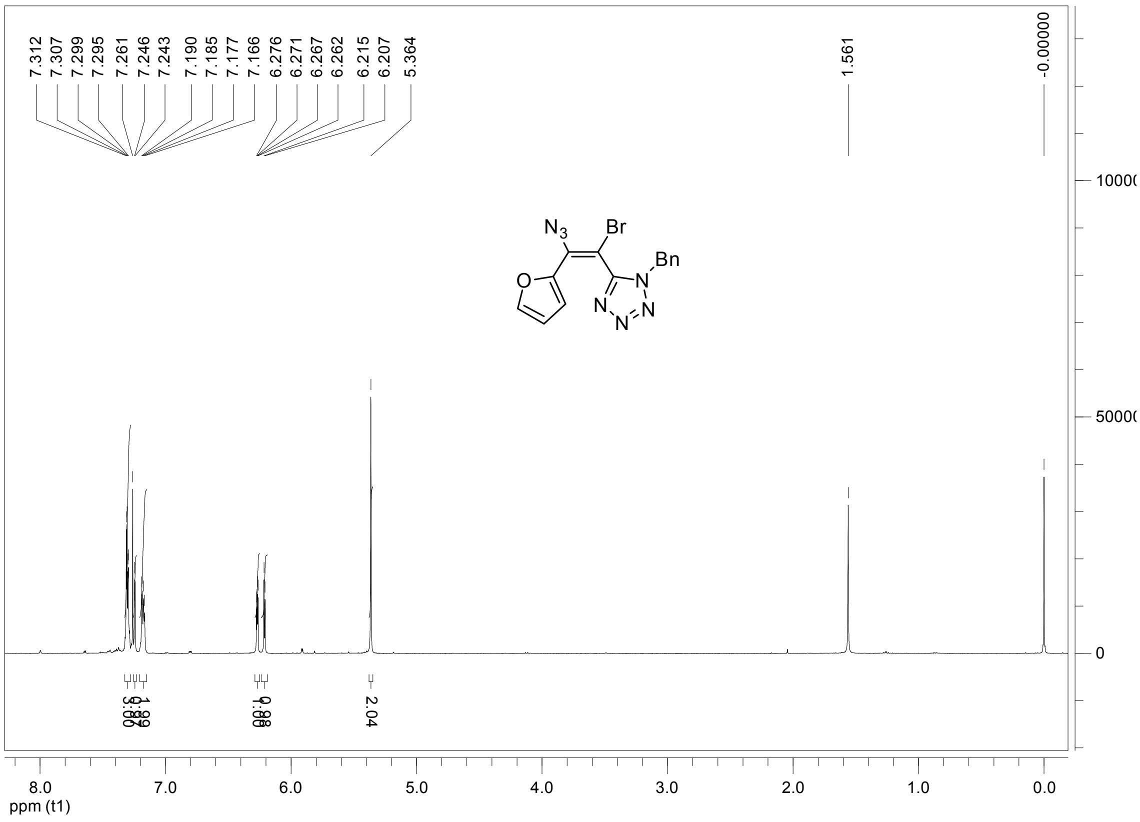

Figure S31. ^{1}H-NMR spectrum of compound **7h**.

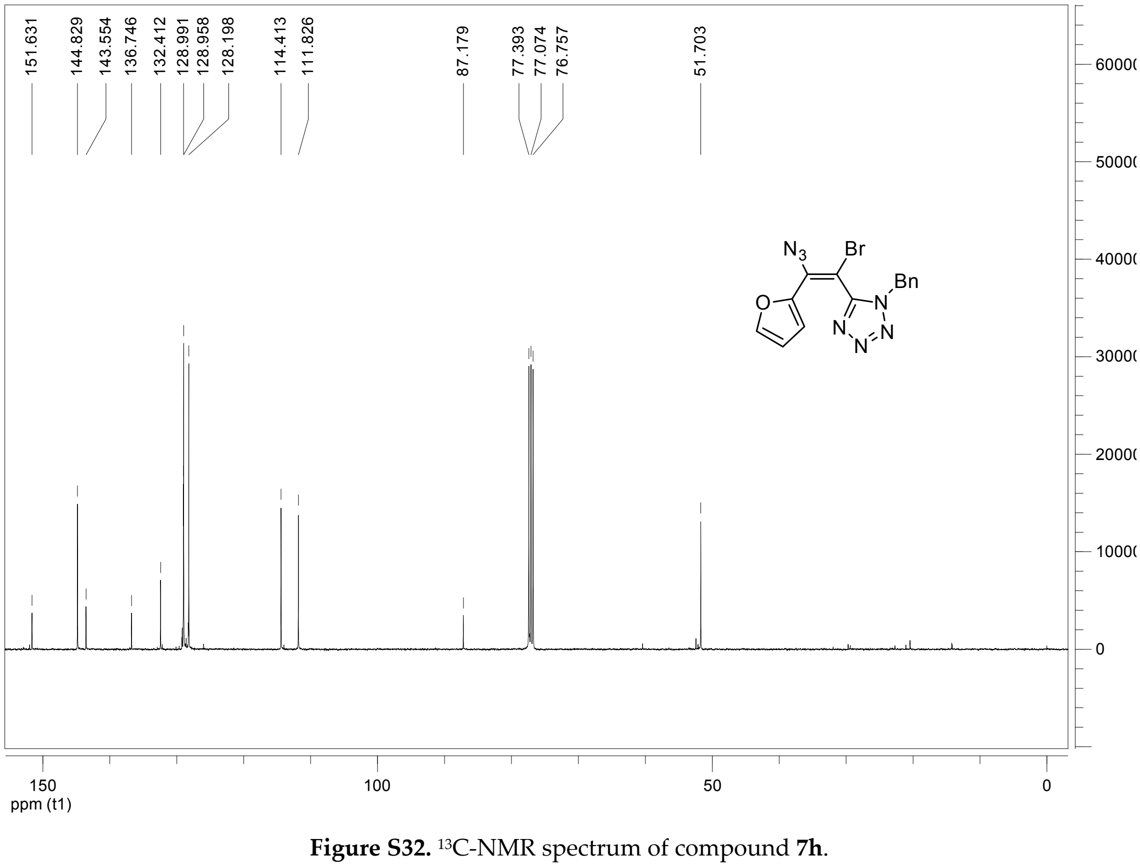

Figure S32. ^{13}C-NMR spectrum of compound **7h**.

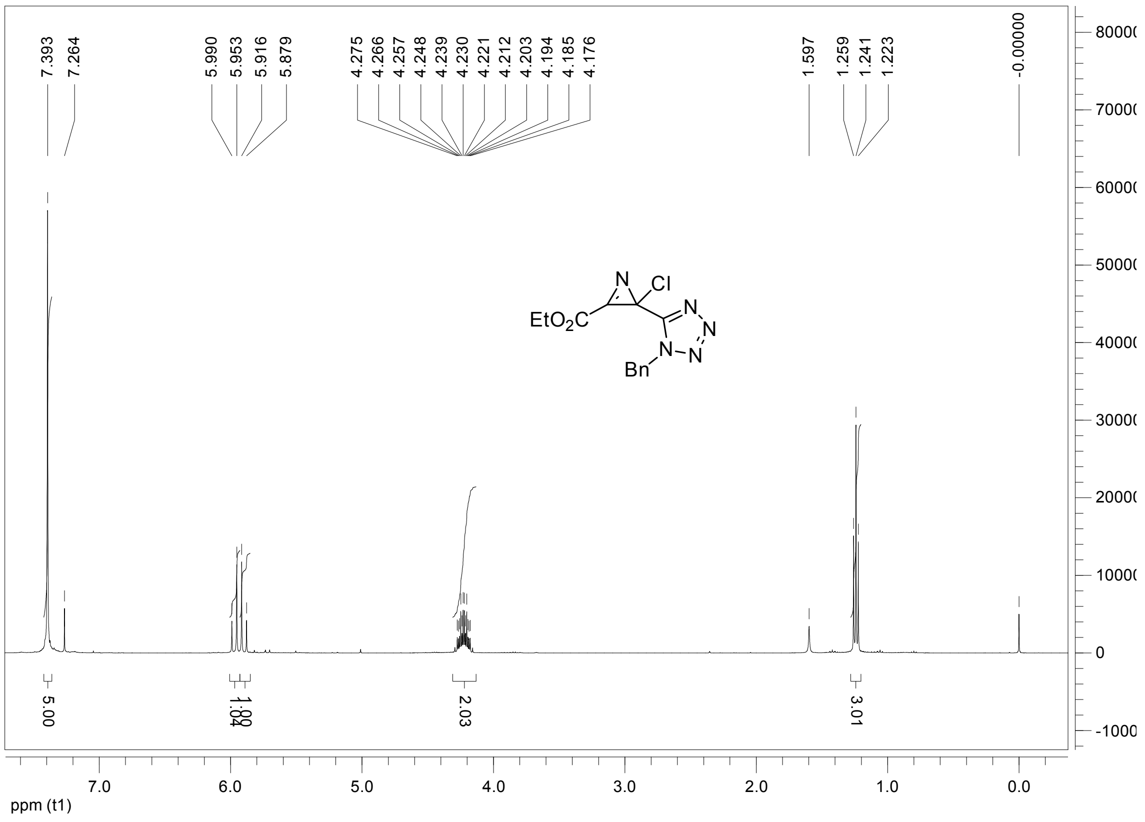

Figure S33. ^{1}H-NMR spectrum of compound **15a**.

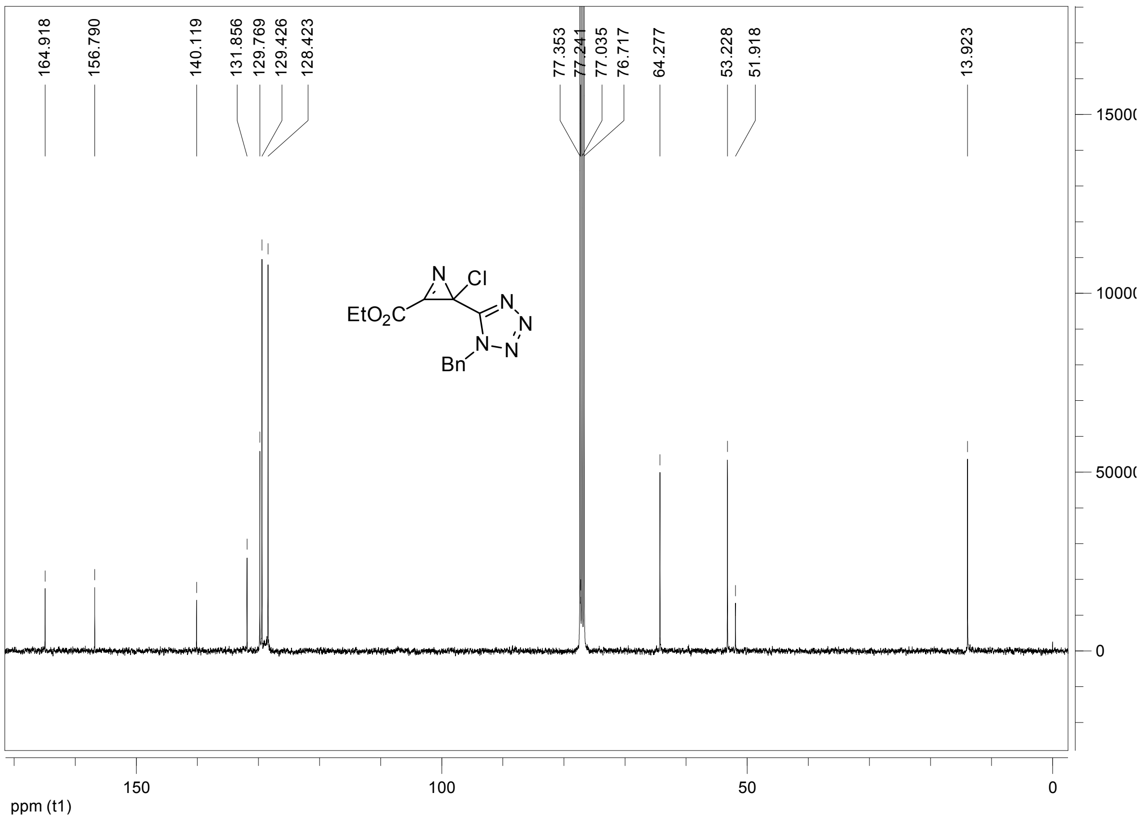

Figure S34. ^{13}C-NMR spectrum of compound **15a**.

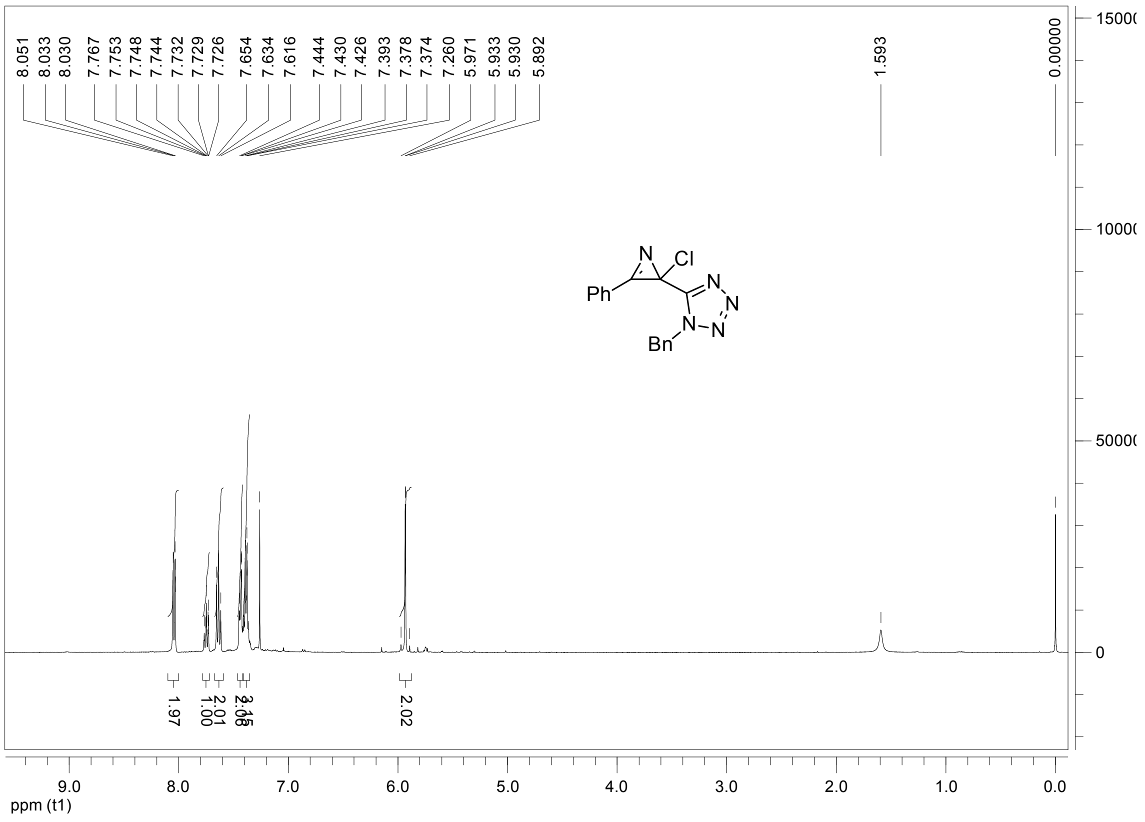

Figure S35. ^{1}H-NMR spectrum of compound **15c**.

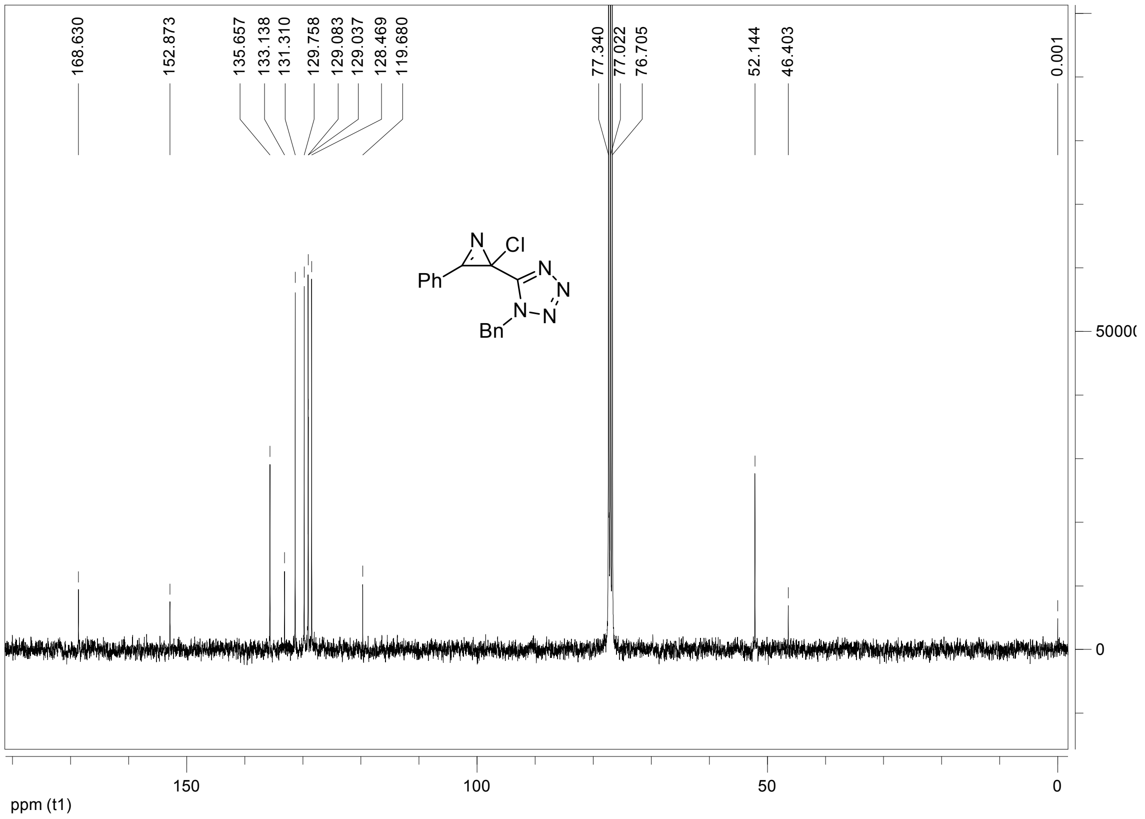

Figure S36. ^{13}C-NMR spectrum of compound **15c**.

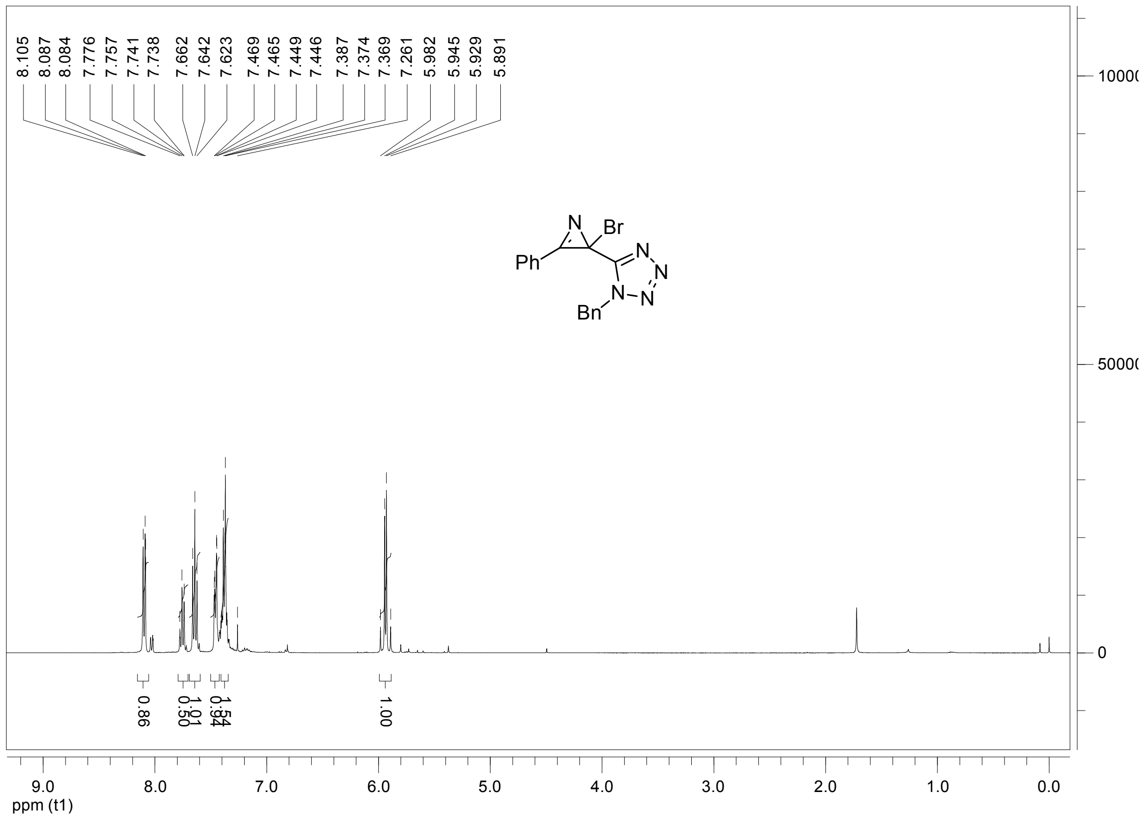

Figure S37. ^{1}H-NMR spectrum of compound **15d**.

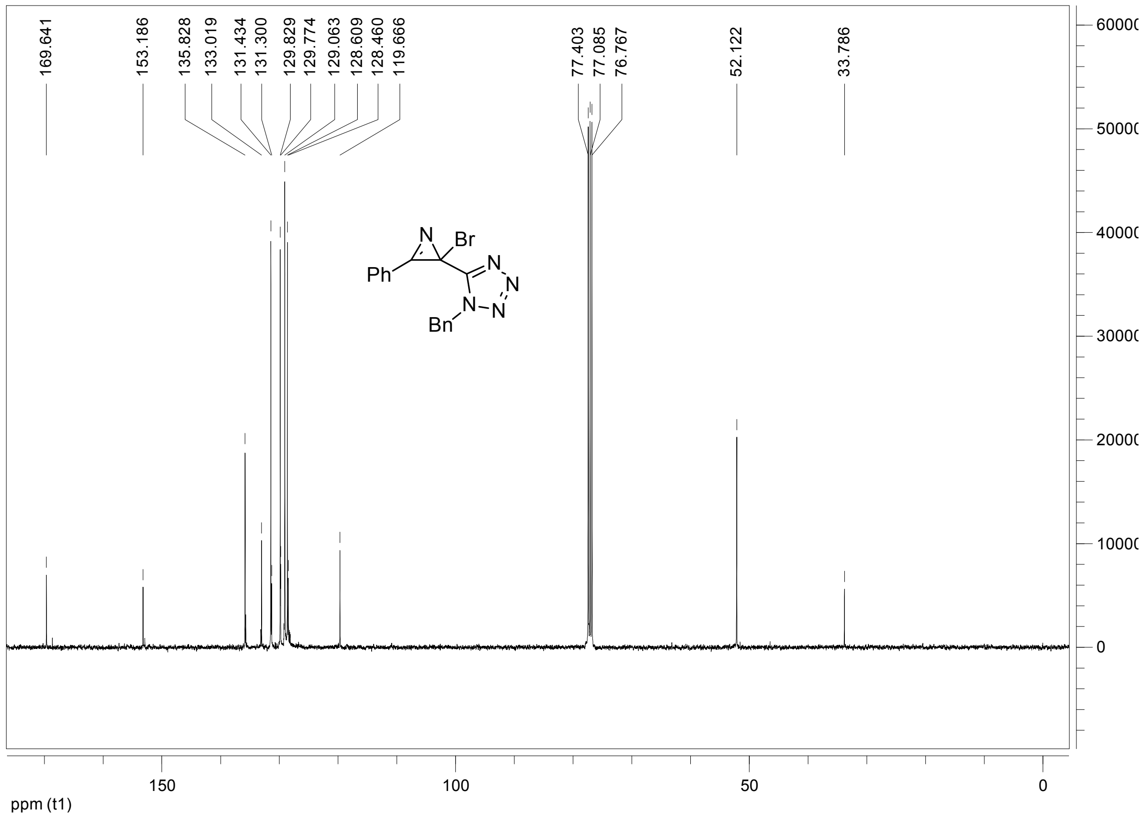

Figure S38. ^{13}C-NMR spectrum of compound **15d**.

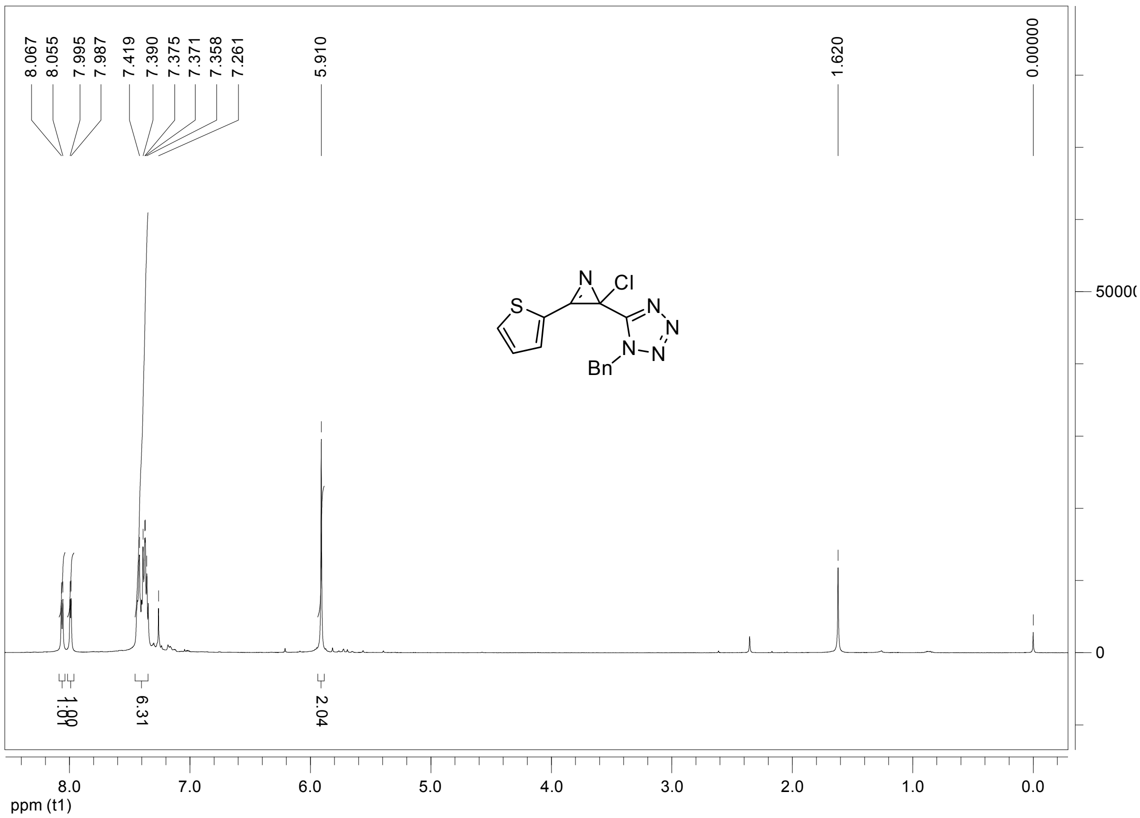

Figure S39. ^{1}H-NMR spectrum of compound **15e**.

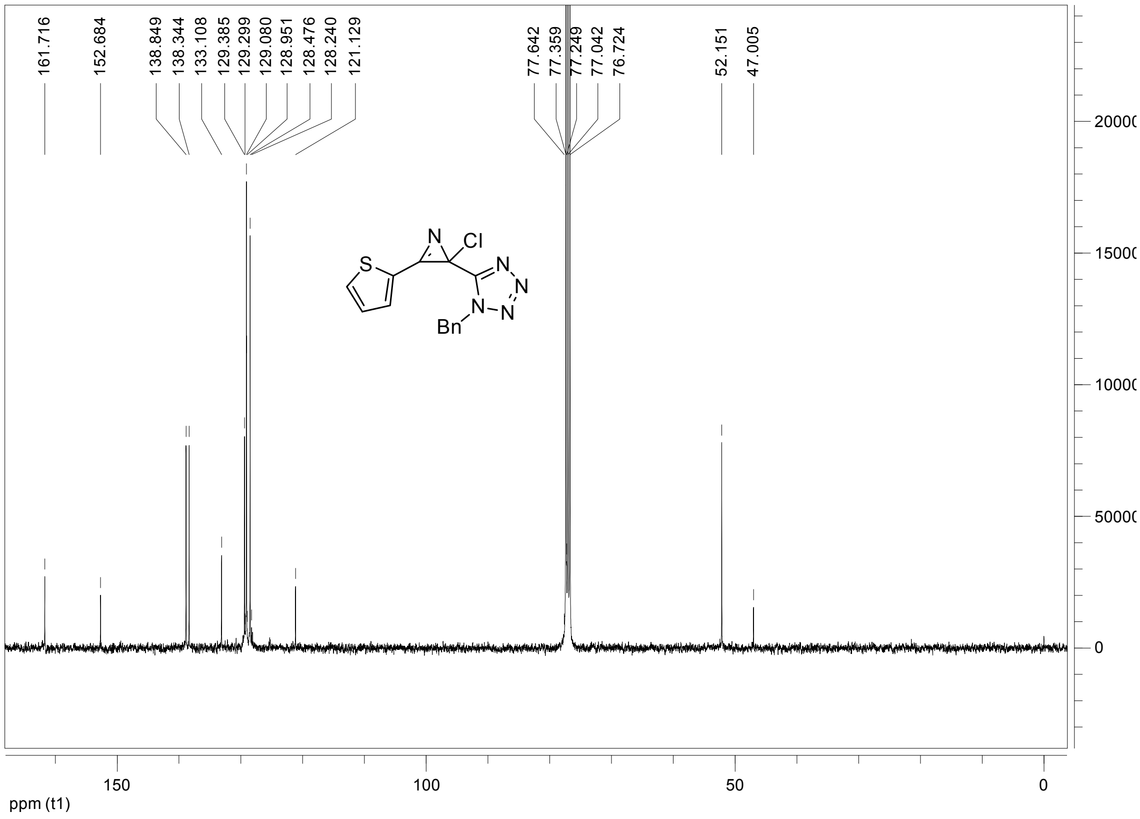

Figure S40. ^{13}C-NMR spectrum of compound **15e**.

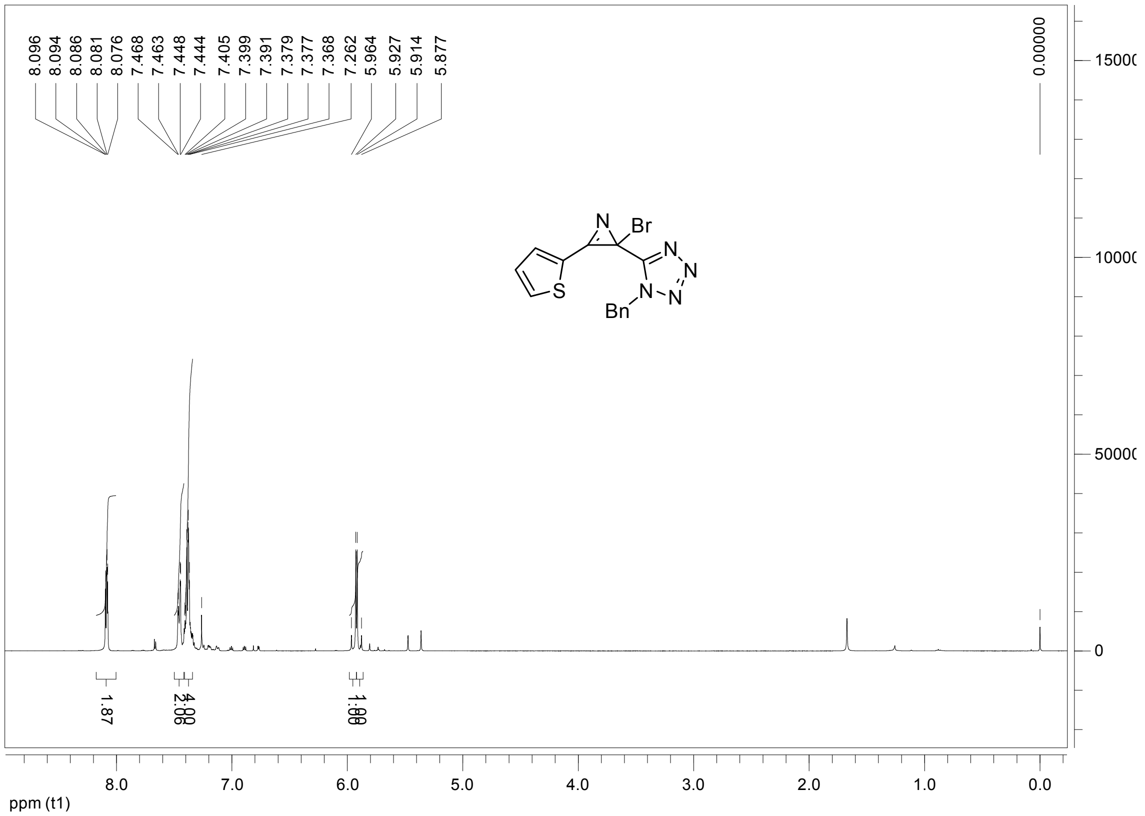

Figure S41. ^{1}H-NMR spectrum of compound **15f**.

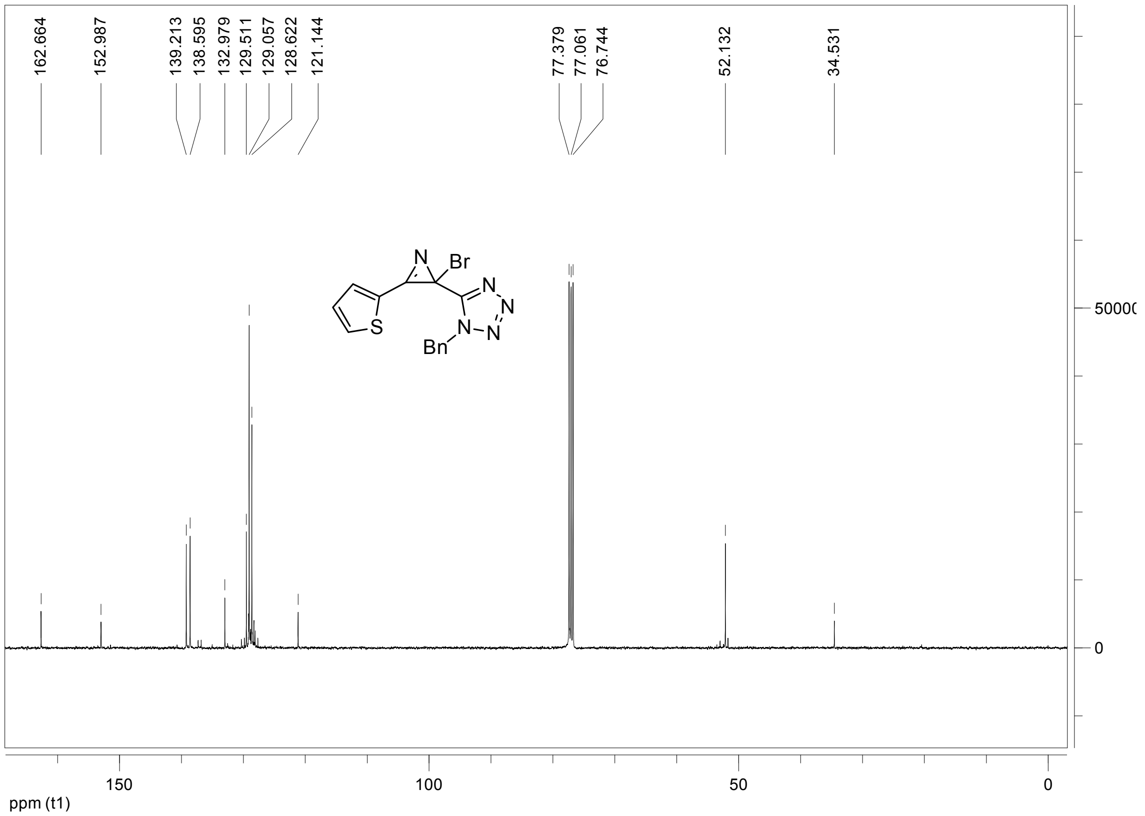

Figure S42. ^{13}C-NMR spectrum of compound **15f**.

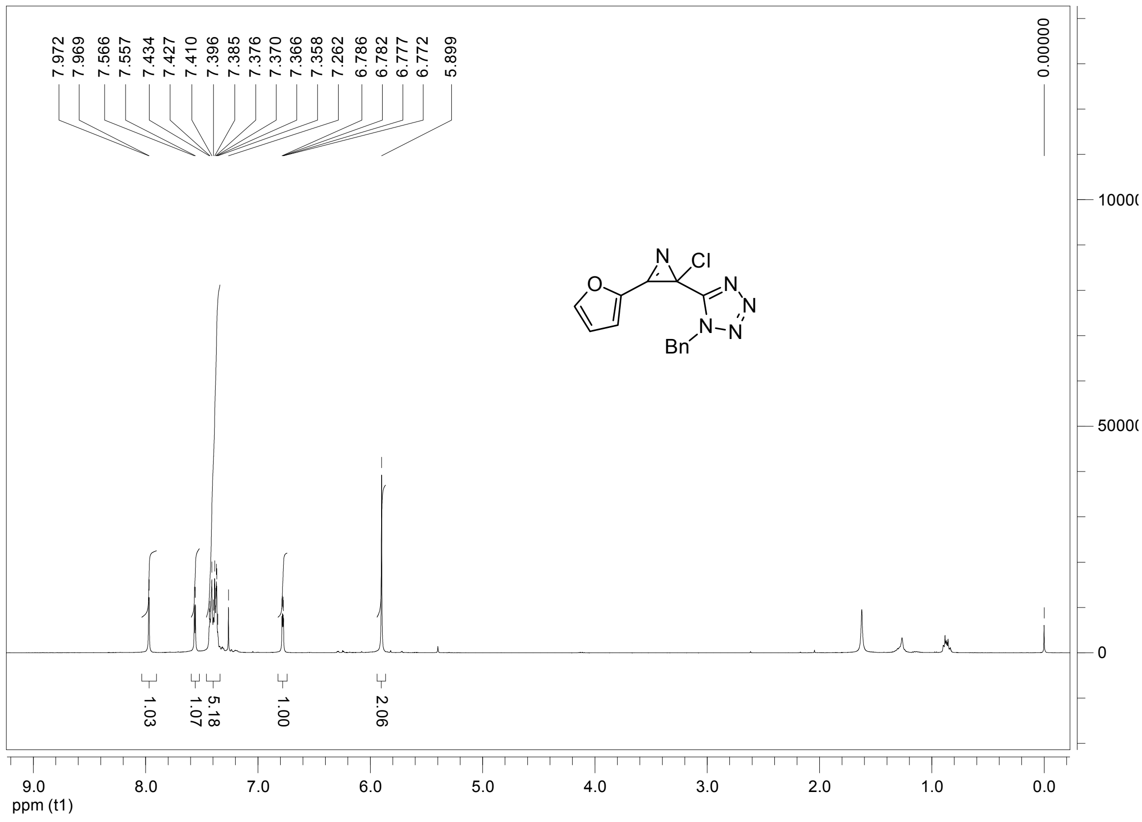

Figure S43. ^{1}H-NMR spectrum of compound **15g**.

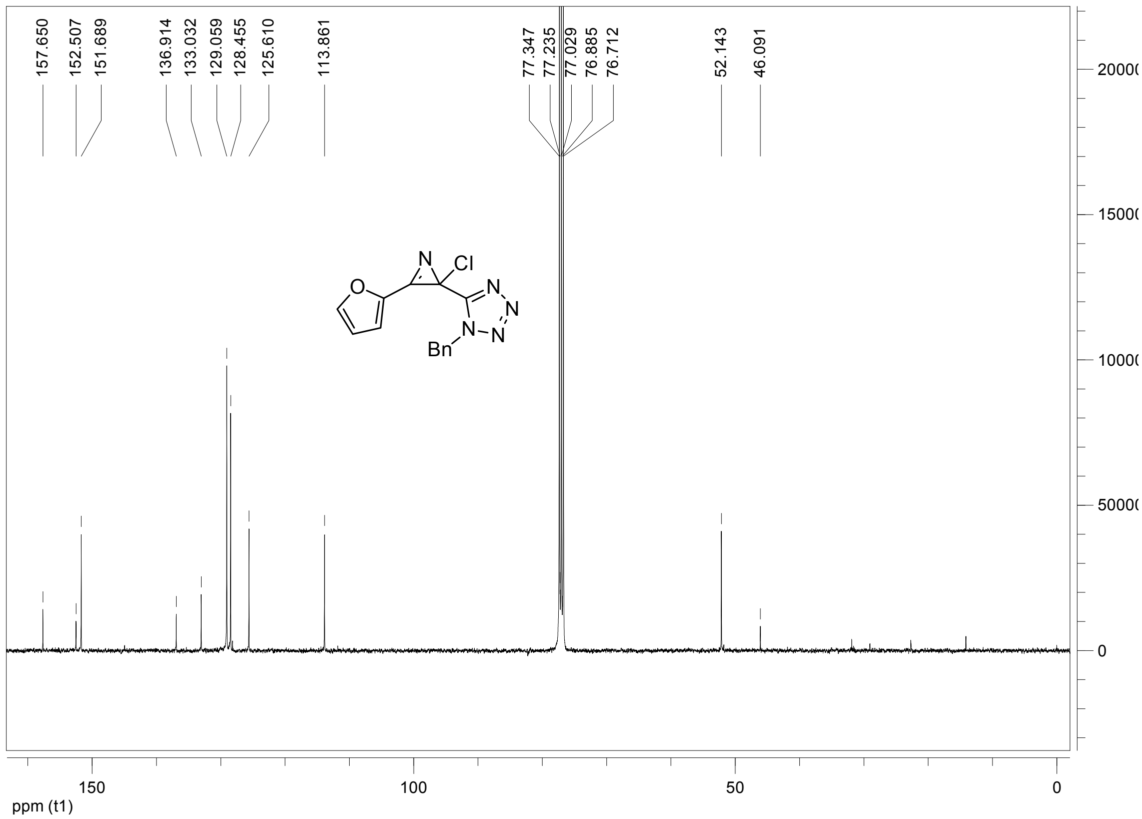

Figure S44. ^{13}C-NMR spectrum of compound **15g**.

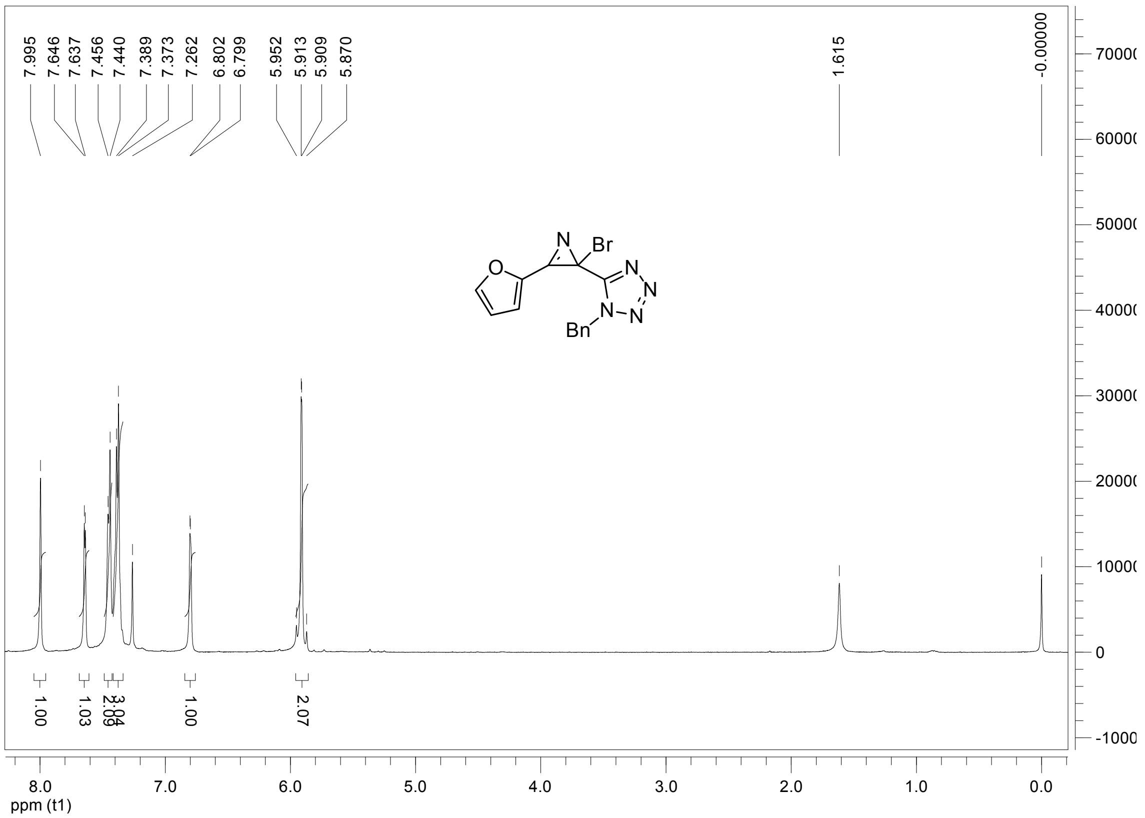

Figure S45. ^{1}H-NMR spectrum of compound **15h**.

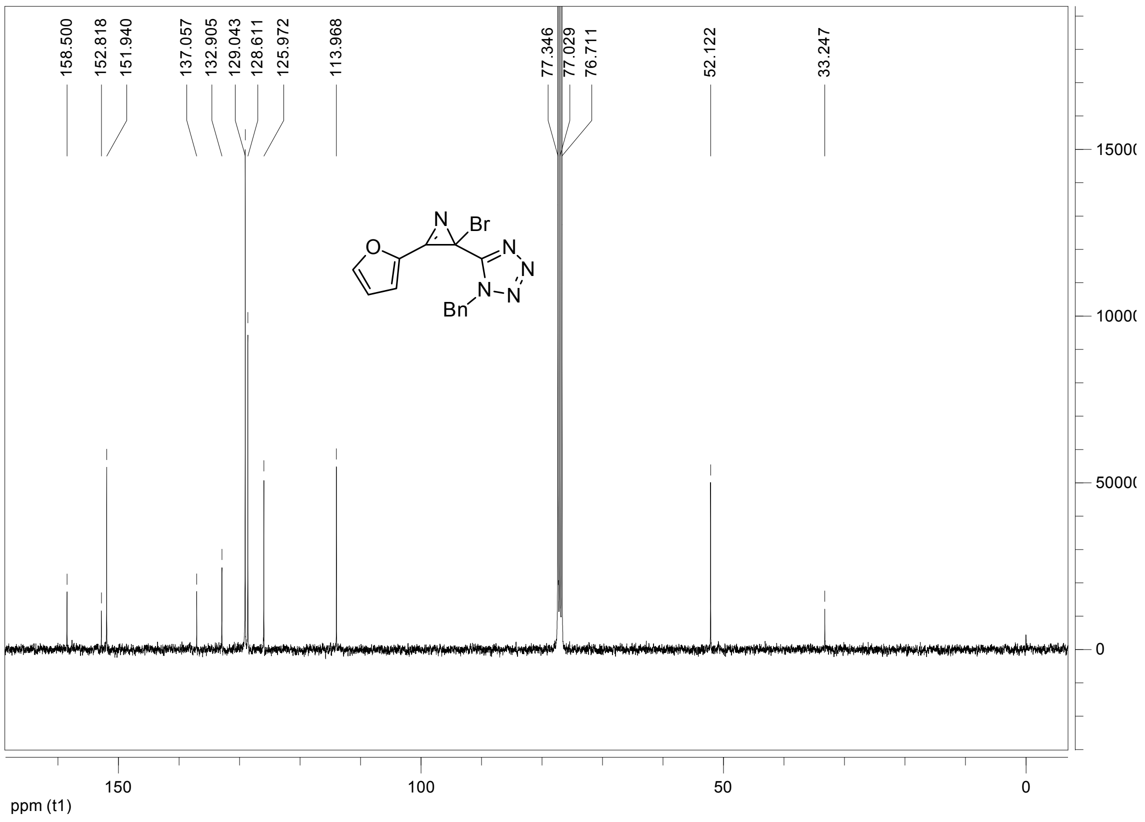

Figure S46. ^{13}C-NMR spectrum of compound **15h**.

2. X-Ray Crystallography Information

Table 1. XRD data collection and refinement details of compounds **7c**, **7h** and **7e**.

	**7c**	**7h**	**7e**
Empirical formula	$C_{16}H_{12}ClN_7$	$C_{14}H_{10}BrN_7O$	$C_{14}H_{10}ClN_7S$
M/g moL^{-1}	337.78	372.20	343.80
Crystal system	monoclinic	monoclinic	monoclinic
Space group	$P2_1/c$	$P2_1/c$	$P2_1/c$
a/Å	15.7997(3)	9.5864(4)	7.8375(2)
b/Å	7.1683(2)	12.3622(5)	23.7676(5)
c/Å	14.8956(3)	14.8024(7)	8.6855(2)
α/°	90	90	90
β/°	98.9320(10)	118.544(3)	105.1090(10)
γ/°	90	90	90
V/ Å^3	1666.57(7)	1540.99(12)	1561.99(6)
Z	4	4	4
D_{calc}/Mg m^{-3}	1.346	1.604	1.462
μ/mm^{-1}	0.242	2.683	0.388
F(000)	696	744	704
Reflections collected	66567	37320	24263
Independent reflections	3836	3533	2745
Restraints/parameters	0/217	0/208	0/227
Goodness of fit on F^2	1.086	1.058	0.971
R_1 (I>2σ_I)	0.0489	0.0457	0.0403
wR_2 (all reflections)	0.1192	0.1322	0.1130
Residual densities	0.172/−0.274	0.986/−0.721	0.176/−0.166

3. ^{1}H-NMR Experiments of 2H-Azirine 15a

The ^{1}H-NMR measurements of compound **15a** were run in DMSO with the original compound which was stored in the refrigerator for 3 months. In this experience the spectra were collected at 25 °C, 35 °C, 45 °C, 55 °C, 75 °C and 95 °C (Figures S48–S53). The ^{1}H-NMR spectrum of compound **15a** freshly prepared is presented in Figure S47.

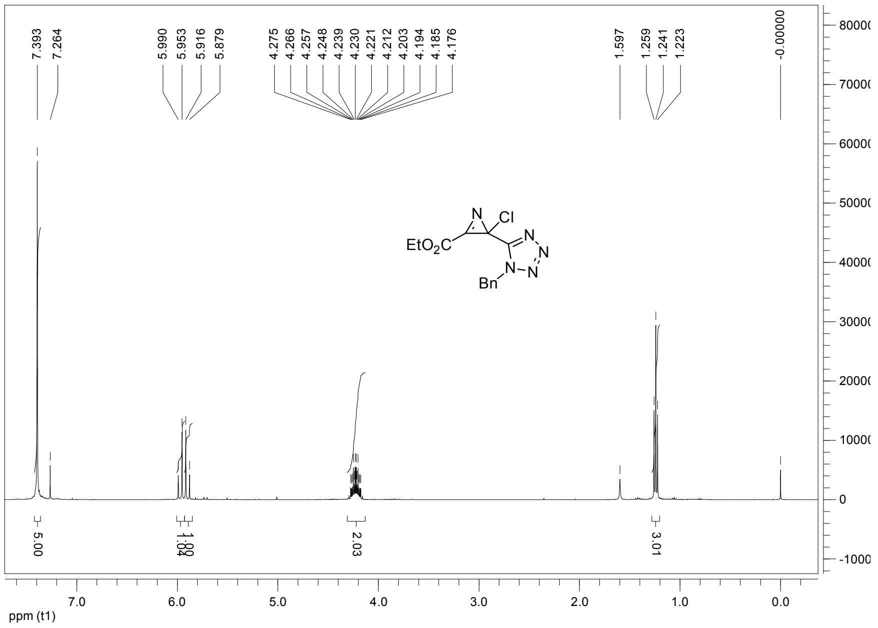

Figure S47. ^{1}H-NMR spectrum of compound **15a** in $CDCl_3$ at 25 °C.

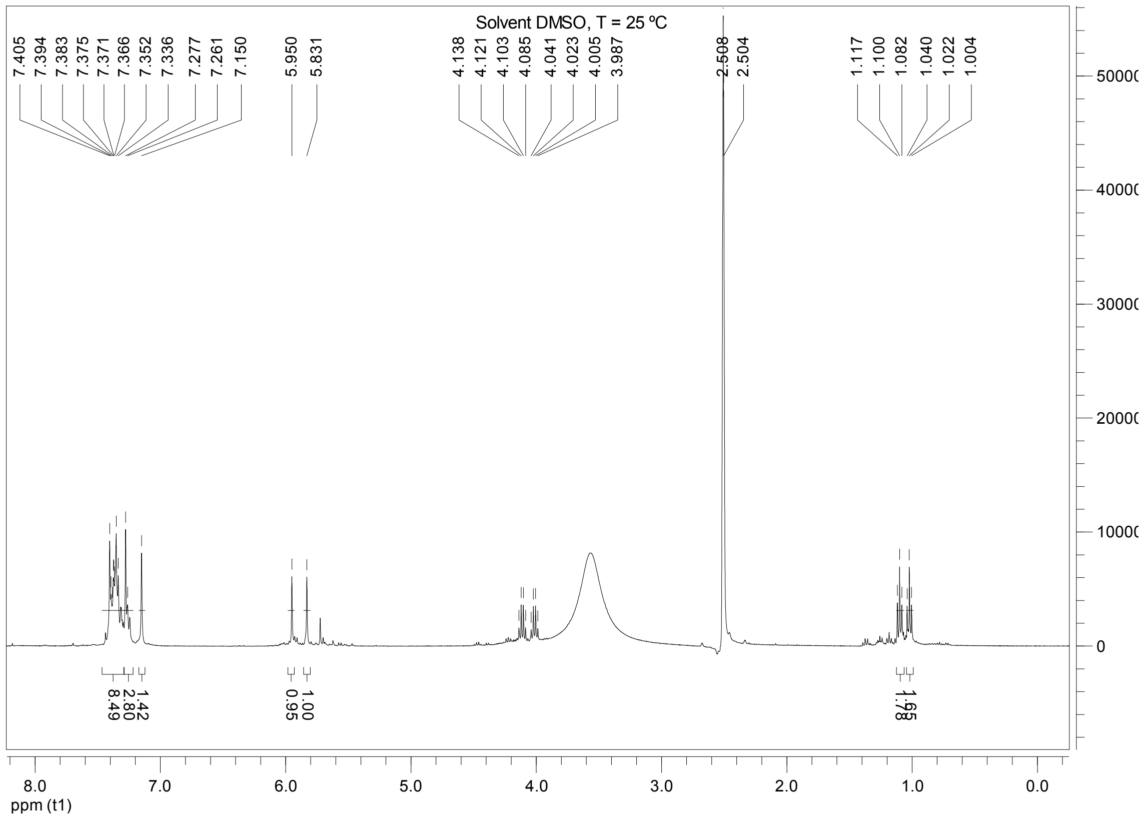

Figure S48. ^{1}H-NMR spectrum of compound **15a** in DMSO at 25 °C.

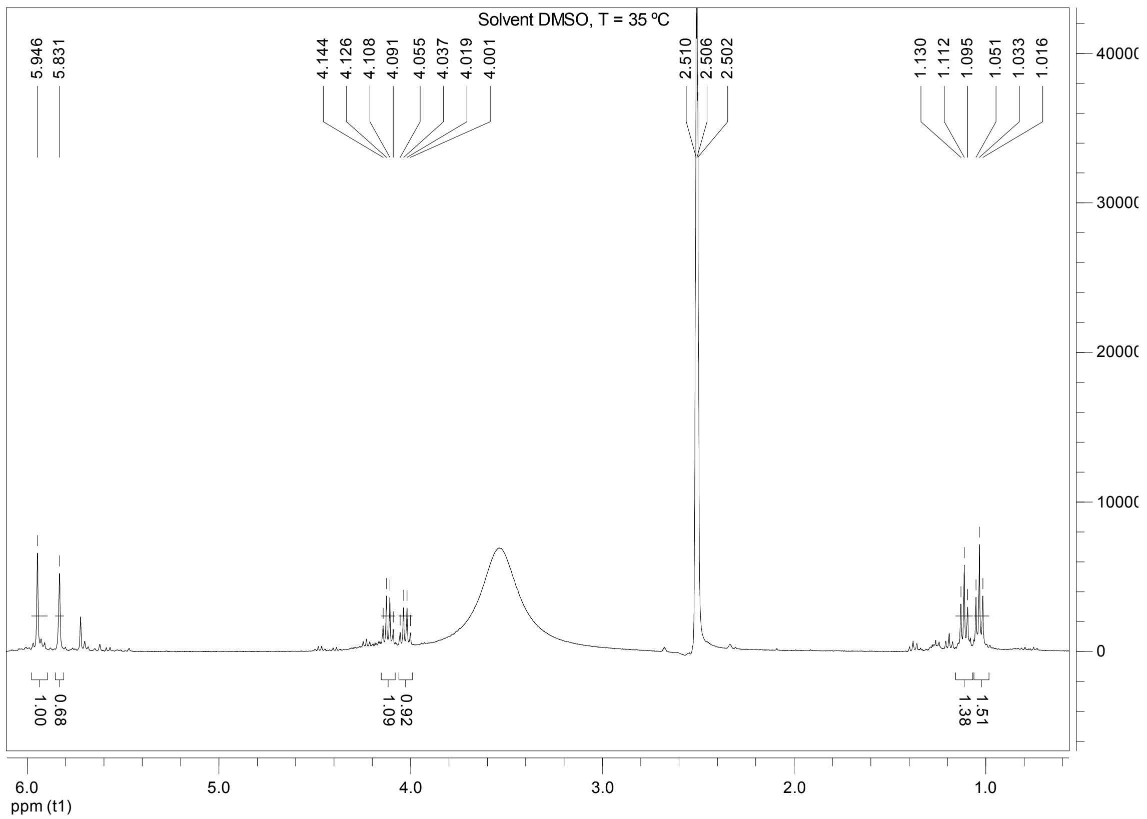

Figure S49. ^{1}H-NMR spectrum of compound **15a** in DMSO at 35 °C.

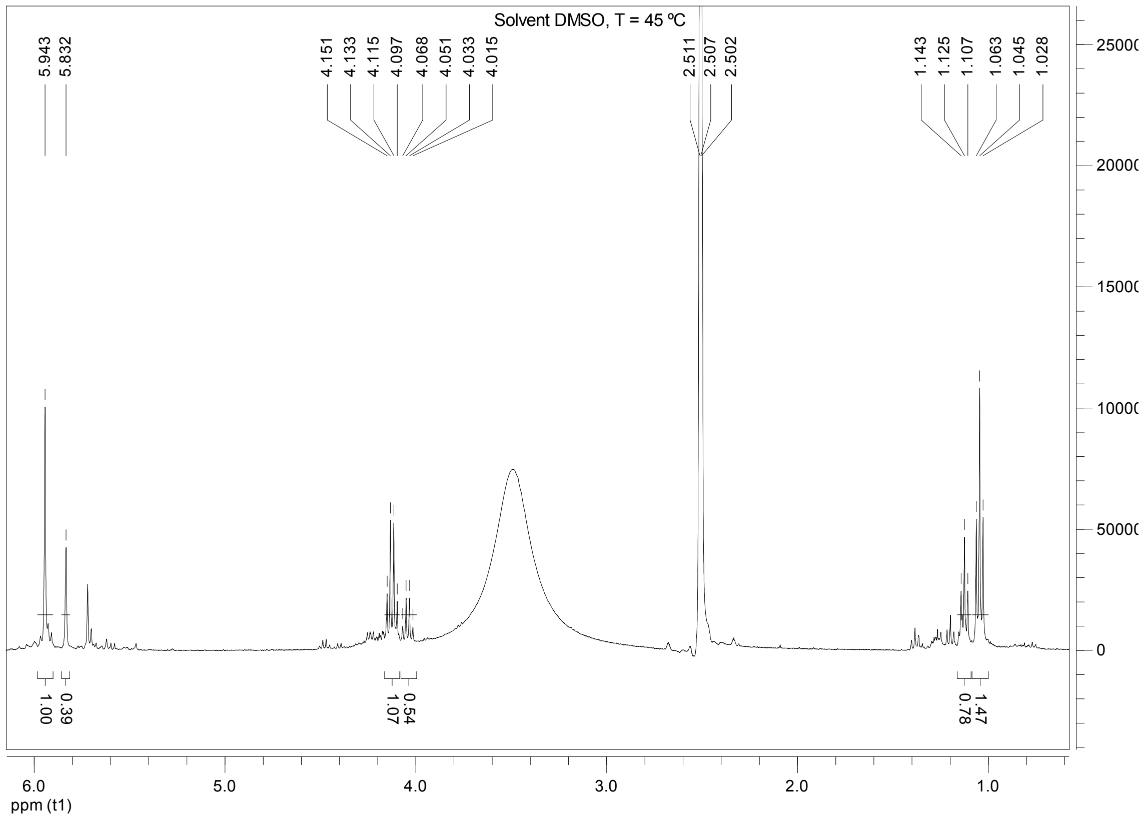

Figure S50. ^{1}H-NMR spectrum of compound **15a** in DMSO at 45 °C.

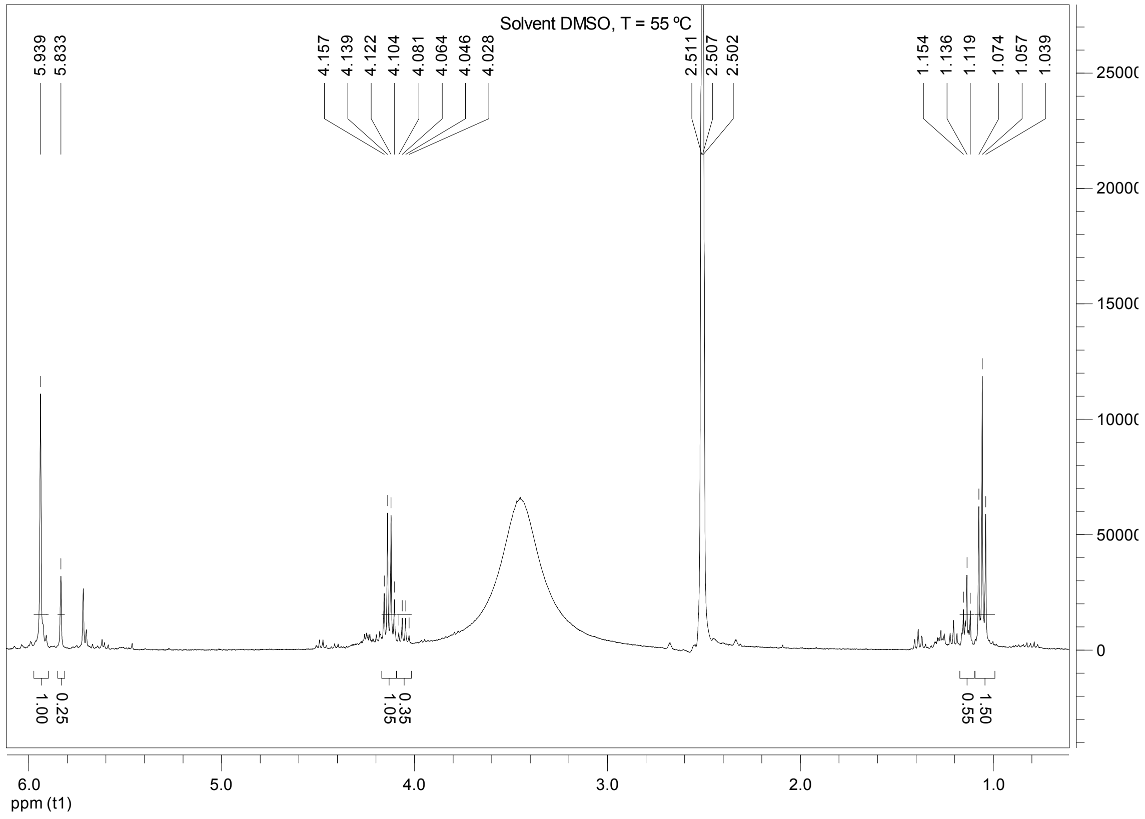

Figure S51. ^{1}H-NMR spectrum of compound **15a** in DMSO at 55 °C.

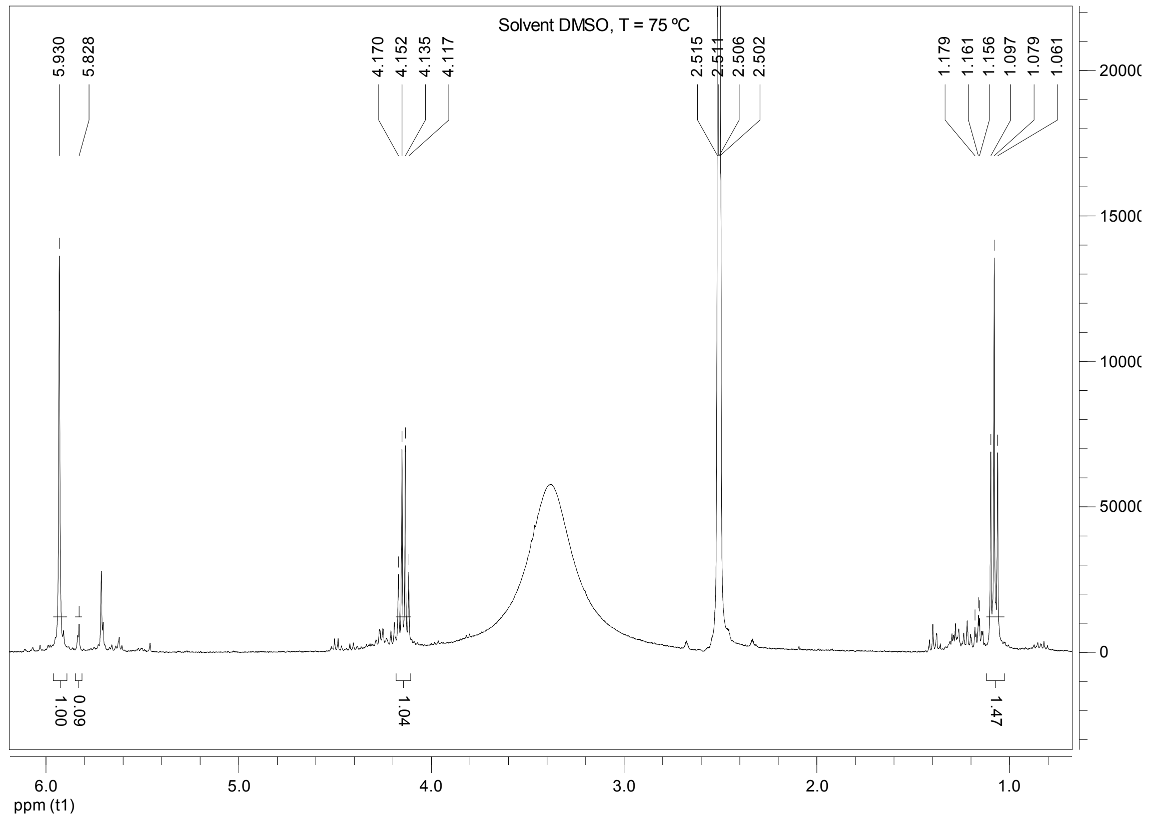

Figure S52. ^{1}H-NMR spectrum of compound **15a** in DMSO at 75 °C.

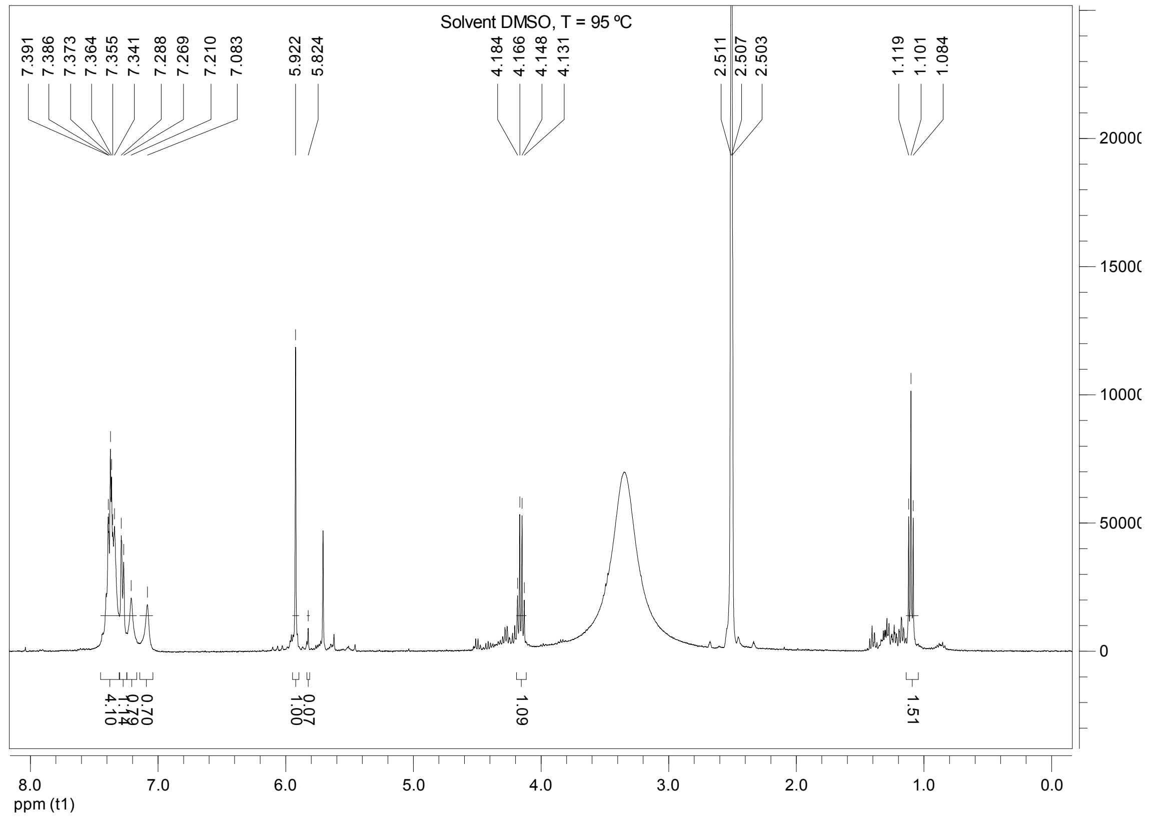

Figure S53. ^{1}H-NMR spectrum of compound **15a** in DMSO at 95 °C.
